# Evidence for independent domestication of sheep mtDNA lineage A in India and introduction of lineage B through Arabian sea route

**DOI:** 10.1038/s41598-021-97761-y

**Published:** 2021-10-05

**Authors:** Ranganathan Kamalakkannan, Satish Kumar, Karippadakam Bhavana, Vandana R. Prabhu, Carolina Barros Machado, Hijam Surachandra Singha, Dhandapani Sureshgopi, Vincy Vijay, Muniyandi Nagarajan

**Affiliations:** 1 Department of Genomic Science, School of Biological Sciences, Central University of Kerala, Kasaragod, Kerala 671316 India; 2 Department of Biotechnology, School of Interdisciplinary and Applied Sciences, Central University of Haryana, Mahendergarh, Haryana 123029 India; 3 Department of Genetic and Evolution, Federal University of São Carlos, Rodovia Washington Luís, Km235 - SP-310, São Paulo, Brazil

**Keywords:** Evolution, Evolutionary genetics, Population genetics

## Abstract

India ranks the second in the world in terms of its sheep population with approximately 74.26 million represented by 44 well-described breeds in addition to several non-descript populations. Genetic diversity and phylogeography of Indian sheep breeds remain poorly understood, particularly for south Indian breeds. To have a comprehensive view of the domestication history of Indian sheep, we sequenced the mitochondrial DNA (mtDNA) control region (D-loop) and cytochrome *b* gene (*CYTB*) of 16 Indian domestic sheep breeds, most of them (13) from the south India. We analysed these sequences along with published data of domestic and wild sheep from different countries, including India. The haplotype diversity was relatively high in Indian sheep, which were classified into the three known mtDNA lineages, namely A, B and C. Lineage A was predominant among Indian sheep whereas lineages B and C were observed at low frequencies but C was restricted to the breeds of north and east India. The median joining network showed five major expanding haplogroups of lineage A (A1–A5). Out of which, A2, A4 and A5 were more frequent in Indian sheep in contrast to breeds from other parts of the world. Among the 27 Indian sheep breeds analysed, Mandya and Sonadi breeds were significantly different from other Indian breeds in the MDS analyses. This was explained by a very high contribution of lineage B into these two breeds. The Approximate Bayesian Computation (ABC) provided evidence for the domestication of lineage A sheep in the Indian subcontinent. Contrary to the current knowledge, we also found strong support for the introduction of lineage B into Indian subcontinent through sea route rather than from the Mongolian Plateau. The neighbour-joining tree of domestic and wild sheep revealed the close genetic relationship of Indian domestic sheep with Pakistani wild sheep *O. vignei blanfordi.* Based on our analyses and archaeological evidences, we suggest the Indian subcontinent as one of the domestication centres of the lineage A sheep, while lineage B sheep might have arrived into India from elsewhere via Arabian sea route. To the best of our knowledge, this is the first comprehensive study on Indian sheep where we have analysed more than 740 animals belonging to 27 sheep breeds raised in various regions of India. Our study provides insight into the understanding of the origin and migratory history of Indian sheep.

## Introduction

Domestic sheep is one of the important livestock species in India and contributes significantly to the livelihood of marginalized small farmers of India. India is the second country with a large population of approximately 74.26 million sheep belonging to 44 described breeds and several non-descript sheep populations^1^. Mitochondrial DNA (mtDNA) studies of sheep breeds across the world have reported two major lineages of A and B, and three minor lineages of C, D and E. The presence of multiple lineages suggests the possibility of their multiple independent domestication events^2–8^. Lineage A, B and C have been identified in Indian sheep with lineage A to be most abundant^1,8–10^. A recent study on the mtDNA control region sequences of Indian sheep proposed that lineage A might have been domesticated in the Indian subcontinent while lineage B might have arrived in the Indian subcontinent through sea route^8^. In contrast, based on the high levels of genetic diversity in sheep breeds of north China and Mongolian Plateau, Lv et al.^11^ suggested that lineage A was brought into the Indian subcontinent from the Middle East via Arabia whereas the lineage B and C entered into the Indian subcontinent from Middle East via Mongolian Plateau. However, these studies^8,11^ have not included south Indian breeds.

Indian sheep breeds have been classified into four major groups based on the agro-ecological regions: (a) north temperate, (b) northwestern arid and semi-arid (c) eastern, and (d) southern peninsular breeds^12^. The south Indian breeds are mostly hairy and primarily have been developed for meat purposes to be easily distinguished from the woolly type of north Indian breeds. The phylogeographic analysis of Indian sheep would be incomplete if the south Indian sheep is not adequately sampled as they contribute nearly 40% to the sheep population of India^12^. Therefore, in the present study, we sequenced the mtDNA D-loop and *CYTB* gene of 16 Indian sheep breeds. Out of these, 13 were from south India while the remaining three from east India. To understand the origin, domestication and migration history of Indian sheep, these new mtDNA sequences were analysed along with published data of sheep breeds of other parts of India and other countries.

## Materials and methods

### Sample collection

**Figure 1:**
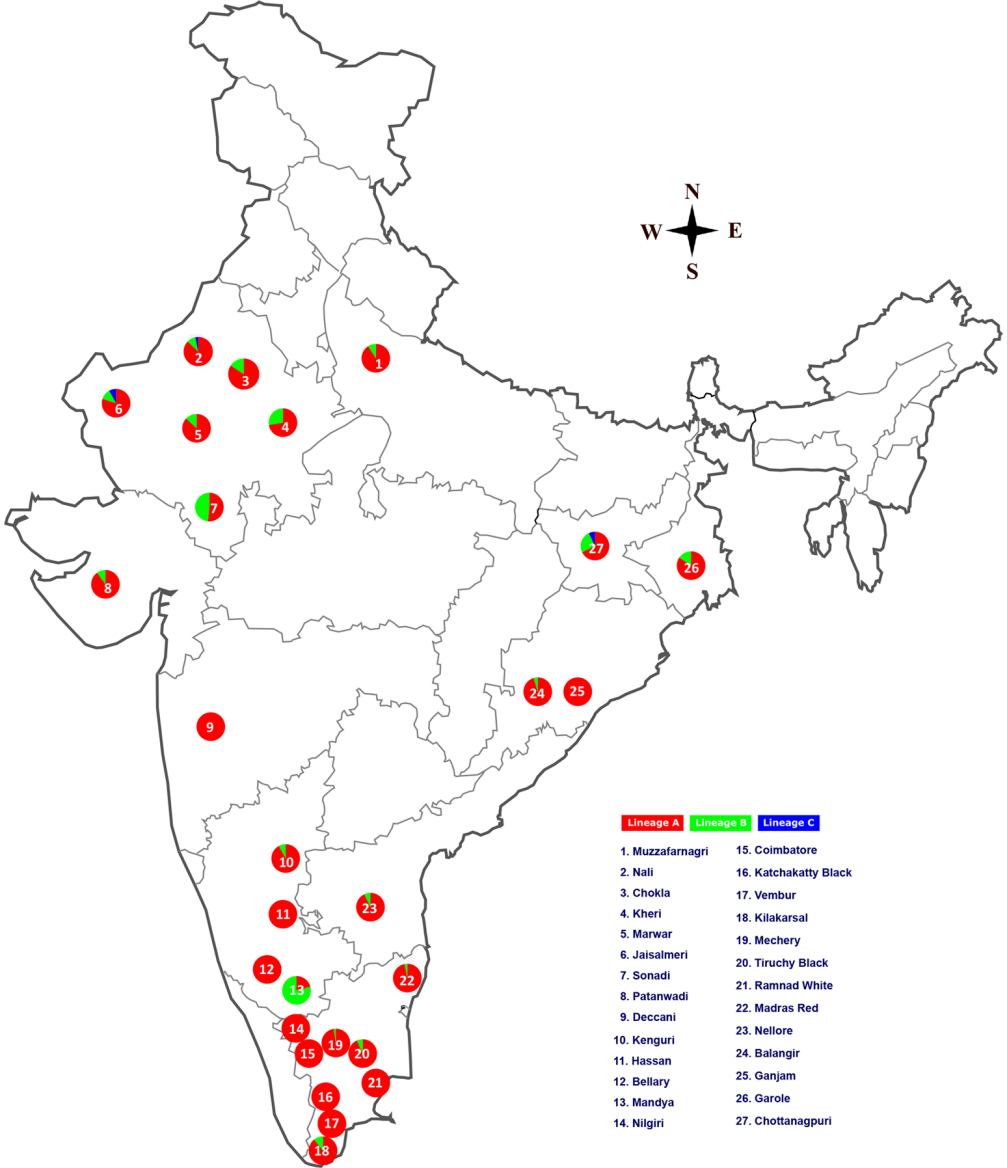
Map showing the sampling locations of sheep breeds and distribution of lineage A, B and C of Indian domestic sheep. The map (source: http://www.d-maps.com/carte.php?num_car=24853&lang=en) was edited in Inkscape 1.0 (https://inkscape.org).

In the present study, 416 animals of 16 Indian sheep breeds, including 13 breeds from south India, were used (Table 1, Fig. 1). The fresh fecal samples were collected from the field/farms within a few minutes of defecation for the purpose of DNA isolation. Sincere efforts were taken to collect only unrelated sheep individuals through interaction with the local herdsmen. The collected feces were stored in absolute ethanol and kept at − 40 °C until DNA extraction. This study used only faecal samples, hence animal ethics committee approval was not required.

**Table 1:** Genetic diversity indices of south Indian sheep breeds.

No.	Breeds	No. of samples		No. of haplotypes		Haplotype diversity	
		D-loop	*CYTB*	D-loop	*CYTB*	D-loop	*CYTB*
1	Bellary	27	30	24	6	0.9886 ± 0.0146	0.4598 ± 0.1086
2	Coimbatore	30	27	22	2	0.9724 ± 0.0172	0.1425 ± 0.0862
3	Hassan	28	27	20	3	0.9735 ± 0.0162	0.2108 ± 0.1005
4	Kenguri	25	28	22	4	0.9900 ± 0.0142	0.3730 ± 0.1065
5	Kilakarsal	28	27	25	2	0.9894 ± 0.0138	0.2051 ± 0.0947
6	Mandya	24	17	12	5	0.8659 ± 0.0544	0.5074 ± 0.1403
7	Madras Red	31	34	28	11	0.9935 ± 0.0100	0.6488 ± 0.0907
8	Katchaikatty Black	6	6	6	3	1.0000 ± 0.0962	0.6000 ± 0.2152
9	Mecheri	41	34	33	10	0.9854 ± 0.0100	0.5437 ± 0.1020
10	Nilgiri	29	29	26	7	0.9926 ± 0.0111	0.6010 ± 0.0974
11	Ramnad White	30	30	19	1	0.9632 ± 0.0167	0.0000 ± 0.0000
12	Tiruchy Black	30	33	23	3	0.9770 ± 0.0163	0.1193 ± 0.0756
13	Vembur	30	30	21	2	0.9747 ± 0.0145	0.0667 ± 0.0613

### DNA extraction, PCR amplification and sequencing

Total genomic DNA was isolated from the feces using QIAamp DNA Stool Mini Kit (Qiagen, Germany) following the procedure of Kamalakkannan et al.^13^. MtDNA D-loop and *CYTB* gene were amplified using the following primer sets 5′-CCAGAGAAGGAGAACAACCAA-3′ and 5′-GCATTTTCAGTGCCTTGCTT-3′; 5′-TGTCATCATCATTCTCACATGG-3′ and 5′-GGGAGGTTGGTTGTTCTCCT-3′ respectively^8^. Amplification was carried out using a thermal cycler with a final volume of 25 µl reaction mixture containing 50 ng of genomic DNA, 12.5 µl of master mix (Promega, Fitchburg, WI) and 2 µl (10 pmol) of each primer under the following PCR conditions: initial denaturation at 94 °C for 5 min, 30 cycles of denaturation at 94 °C for 1 min, annealing at 50 °C for D-loop or 53 °C for *CYTB* gene for 1 min, extension at 72 °C for 1 min and the final extension at 72 °C for 5 min. Eventually, the PCR products were purified and both strands were sequenced using Big Dye Terminator Cycle sequencing kit (Applied Biosystems, USA) on ABI 3500 Genetic Analyzer (Applied Biosystems, USA) as per the manufacturer’s instructions.

### Genetic diversity and phylogenetic analysis

The generated D-loop (GenBank accession numbers: MZ090959–MZ091374) and *CYTB* gene (GenBank accession numbers: MZ216042–MZ216452) sequences were edited and aligned using the MEGA (version 7) software^14^. Various population genetics parameters like number of haplotypes, haplotype diversity, AMOVA, pairwise F_ST_ values and mismatch distribution were computed using ARLEQUIN 3.1^15^. The calculated pairwise F_ST_ values were displayed on the multidimensional scaling (MDS) plot using R program (www.r-project.org). Neighbour-joining (NJ) tree was constructed using MEGA (version 7)^14^ with 2000 bootstrap and maximum composite likelihood as substitution model to categorize the new sequences based on the reference lineage sequences (HM236174, HM236177, HM236178, HM236180 and HM236182) retrieved from GenBank^16^. The median-joining (MJ) network was prepared using the program Network 10.2.0.0^17^.

### Bayesian inference of sheep domestication history

**Figure 2:**
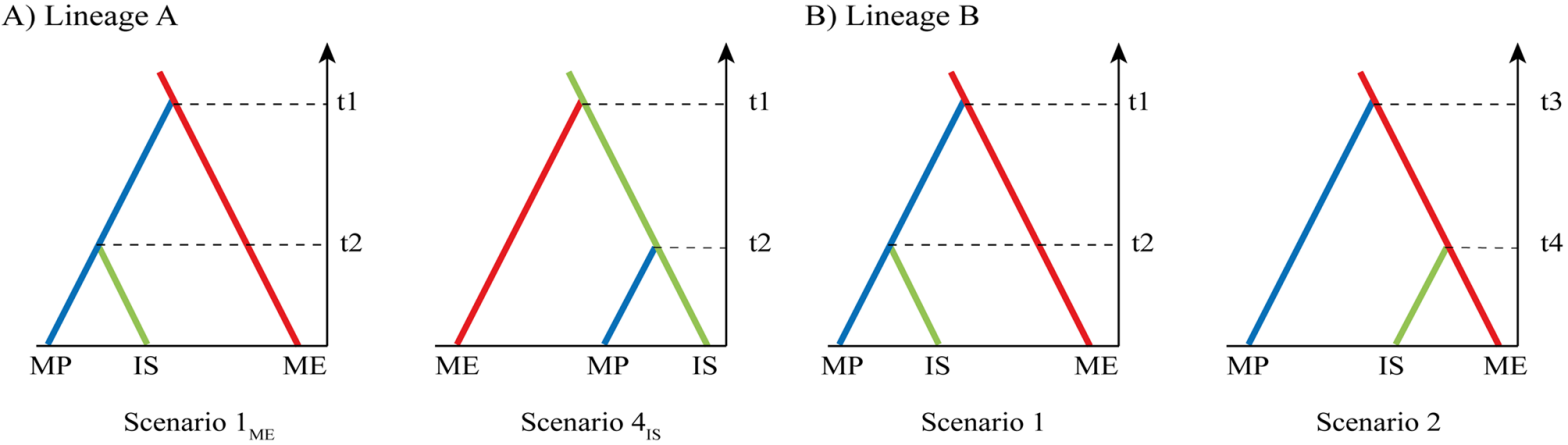
Colonization scenarios tested using DIYABC v2.1 in (**A**) lineage A and (**B**) lineage B of domestic sheep. Colours represent the populations: ME (the Middle East, red), MP (the Mongolian Plateau, blue) and IS (the Indian subcontinent, green). In lineage B, the second scenario has a different time divergence designation because it is unknown which lineage diverged first from the Middle East.

To better understand the colonization history of domestic sheep lineages, we employed the approximate Bayesian computation (ABC) statistical approach to test different hypotheses of dispersal routes using the D-loop sequences. Simulations were conducted in DIYABC v2.1 software^18^. The scenarios were built based on the obtained results and previous study^11^. For lineages of A and B, we considered three metapopulations according to their geographical distributions: The Middle East (ME), the Mongolian Plateau (MP) and the Indian subcontinent (IS). For lineage A, our question was about its origin and colonization routes. To test our hypotheses, we adopted a stepwise procedure to decrease the computational time. In the first round, we built two independent models each with four scenarios, considering the source population identity as the main difference between them (ME and IS, Supplementary Fig. S1). The lineage A scenarios considering ME (named here A_ME_ scenarios) as the source were: (1_ME_) lineage A spread first from ME to MP at time t1, and posteriorly from MP to IS at time t2; (2_ME_) lineage A expand from ME to IS at time t1, then from IS to MP at time t2; the scenarios 3_ME_ and 4_ME_ occur through two independent colonization events from ME, first MP at time t1 and second IS at time t2 (scenario 3_ME_), while in the scenario 4_ME_ we considered the first colonization to IS and then to MP. The lineage A scenarios considering IS (hereafter A_IS_ scenarios) as the source were: (1_IS_) lineage A spread first from IS to MP at time t1, and posteriorly from MP to ME at time t2; (2_IS_) lineage A expand from IS to ME at time t1, then from ME to MP at t2; in the scenarios 3_IS_ and 4_IS_, IS was the source population for both MP and ME. In the scenario 3_IS_ the first colonization was to MP and then to ME, while in the 4_IS_ scenario the lineage first spread to ME, and then to MP. The scenarios with high posterior probabilities in each model were tested in the second round (Fig. 2A). For lineage B, we were interested to know the colonization route for the Indian subcontinent (Arabian sea route or Inland route). Two scenarios were hypothesized: (1) lineage B spread from ME to MP at time t1, and posteriorly from MP to IS at time t2 (Inland route) as previously suggested by Lv et al.^11^; (2) the IS lineage B was originated from ME in an independent colonization event (Arabian sea route) (Fig. 2B). Prior distributions of demographic, historical and mutations parameters are summarized in Supplementary Table S2. For each lineage, we set the mutation model according to its substitution model estimated in JModelTest^19^. The mean mutation rate was assigned as 10^–8^ to 10^–7^ per site per generation^20^. The statistical summaries (SS) were chosen after we performed a principal components analysis to pre-evaluate the similarity between simulation datasets and the empirical data (data not shown). The SS retained for the generation of the simulated dataset in both lineages were mean of pairwise differences, mean of number of rarest nucleotides at segregating sites, and variance of numbers of the rarest nucleotides at segregating sites, all are considering one-sample SS. Two-sample SS included mean of pairwise differences (B) and F_ST_.

After simulating one million datasets for each competing scenario, we performed a principal components analysis to pre-evaluate the similarity between simulation datasets and the empirical data. The posterior probability for each scenario were assessed with polychotomous weighted logistic regression^20^ on the 1% of simulated datasets that were closest to the observed data. Winner scenarios had the highest significant posterior probability value with a nonoverlapping 95% confidence interval (CI). To evaluate the confidence of the winner scenario, the posterior predictive error was calculated using 1000 simulated datasets in the logistic approach. Parameter estimation was conducted for the scenario with high posterior probabilities using local linear regression^21^ on the 1% closest simulated datasets, applying logit transformation to all parameter values. The precision of each parameter estimation was evaluated by calculating the relative median of the absolute error (RMAE)^20^. Finally, we assessed a model verification step by evaluating the goodness-of-fit of the winner scenario concerning the observed dataset.

## Results

### Genetic diversity of south Indian sheep breeds

#### D-loop

A total of 359 mtDNA D-loop sequences were generated from 13 south Indian sheep breeds. The 950 bp D-loop sequences revealed 233 haplotypes which resulted from 101 polymorphic sites. Of the 233 haplotypes, 180 were unique and the most frequent haplotype was present in 22 individuals from 10 breeds. The haplotype diversity ranged from 0.8659 ± 0.05 in Mandya breed to 1.0 in Katchaikatty Black breed with only six samples (Table 1). Besides Katchaikatty Black, Madras Red had the second highest haplotype diversity (0.9935 ± 0.01). The AMOVA showed 26.44% of variation among the south Indian breeds. Further, the calculated pairwise F_ST_ values were plotted on MDS where all the south Indian sheep breeds formed a single cluster except Nilgiri and Mandya breeds (Supplementary Fig. S2A). To determine whether the high variation among the breeds was due to the effect of Mandya and Nilgiri breeds, the AMOVA was reperformed in three different combinations (1) Both Mandya and Nilgiri breed were excluded, (2) Only Nilgiri breed was excluded and (3) Only Mandya breed was excluded. The variation among breeds remained to be similar in combination 1 and 2. However, in combination 3, the variation among breeds was reduced down to 7.22%, suggesting Mandya breed to be distinct from other south Indian breeds.

#### CYTB gene

A total of 352 mtDNA *CYTB* gene sequences were generated from 13 south Indian sheep breeds. The 856 bp *CYTB* gene showed 36 haplotypes defined by 25 polymorphic sites. The maximum haplotype diversity was found for the Madras Red breed (0.6488 ± 0.0907) while only one haplotype was present among 30 Ramnad White sheep sampled from different flocks and places. The AMOVA showed 24.11% of variation among the south Indian breeds. Further, the pairwise F_ST_ values were plotted on MDS which showed Mandya breed to be distinct from others (Supplementary Fig. S2B). Based on the MDS result, the AMOVA was repeated by excluding Mandya breed. Similar with the D-loop data, the variation among breeds was decreased to 10.09%, confirming the unique maternal lineage of Mandya breed compared with other south Indian breeds.

### Phylogenetic structure of south Indian sheep breeds

**Figure 3:**
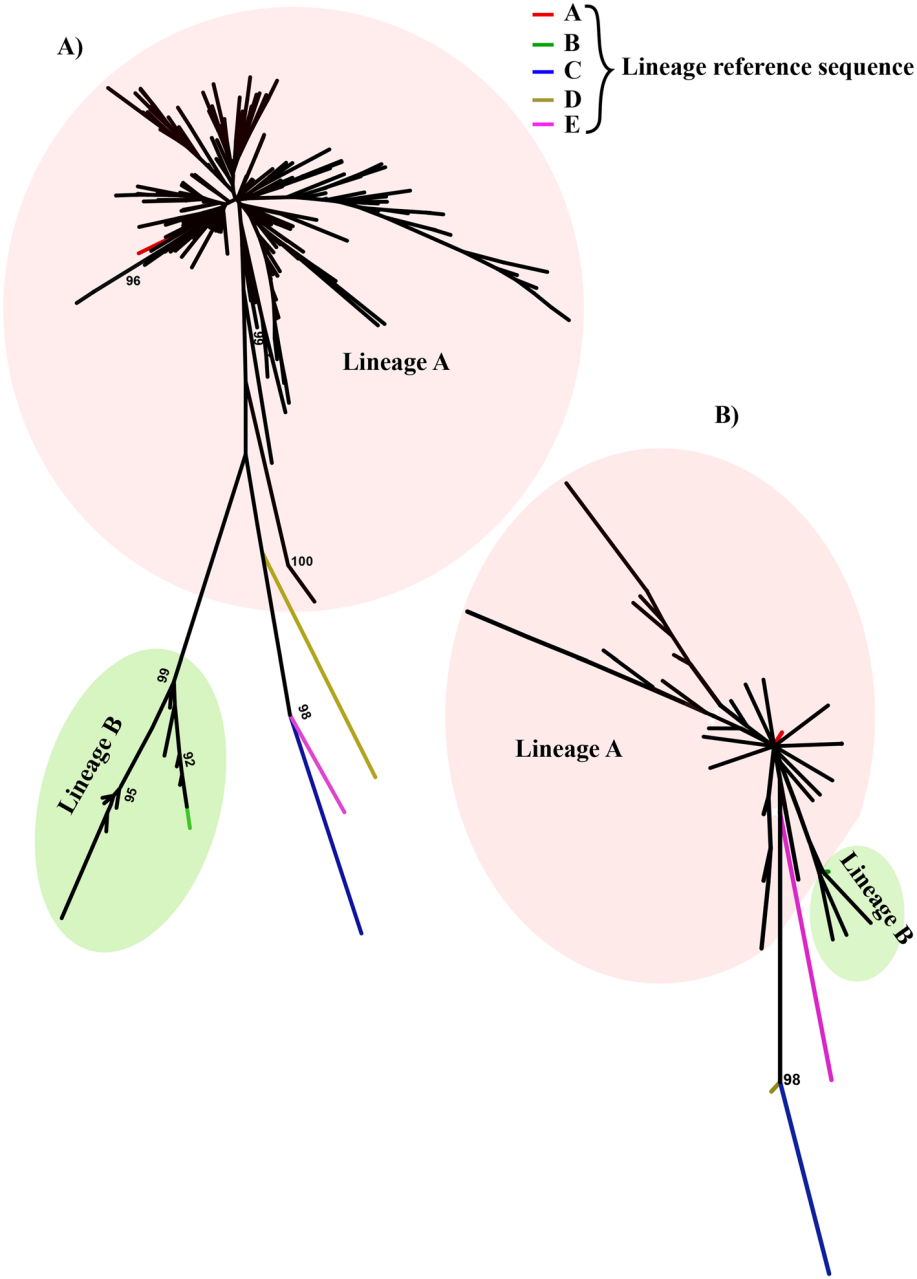
Neighbour-joining phylogenetic tree of south Indian domestic sheep. The NJ tree was based on 359 and 352 sequences for (**A**) D-loop (950 bp) and (**B**) *CYTB* gene (856 bp), respectively. The NJ tree was constructed using the software MEGA (version 7)^14^ and the final output was edited in Inkscape 1.0 (https://inkscape.org).

To understand the phylogenetic relationship of south Indian sheep breeds, we constructed NJ trees using D-loop and *CYTB* gene sequences along with representative lineage sequences. The NJ trees showed two distinct major clades for south Indian sheep which corresponded to the lineages of A and B (Fig. 3). The lineage A was found to be predominant, which consisted of 92.2% of south Indian sheep while the lineage B consisted of only 7.8% of south Indian sheep.

Median-joining networks were constructed using D-loop and *CYTB* gene sequences to understand the distribution pattern of south Indian sheep haplotypes. The MJ network of D-loop was found to be complex but clearly distinguished the lineages of A and B (Supplementary Fig. S3). The lineage A was predominant in all the breeds except Mandya. Notably, seven breeds of Bellary, Coimbatore, Hassan, Katchaikatty Black, Nilgiri, Ramnad White and Vembur were fully encompassed with lineage A while Kenguri, Kilakarsal, Madras Red, Mecheri and Tiruchy Black breeds all had very low occurrences (0.28–0.84%) of lineage B. In contrast, majority of the Mandya sheep carried lineage B (79%). Lineage A displayed five major expanding haplogroups (A1, A2, A3, A4 and A5), indicating extensive expansion of lineage A among south Indian sheep. Also, there was a large number of singletons in the lineage A. On the other hand, lineage B showed no star like expansion among south Indian sheep breeds, indicating the lack of expansion of lineage B. There was no difference between the MJ networks of *CYTB* gene and D-loop haplotypes, other than the complexity of network (Supplementary Fig. S4).

### Phylogeography of Indian sheep breeds

**Figure 4:**
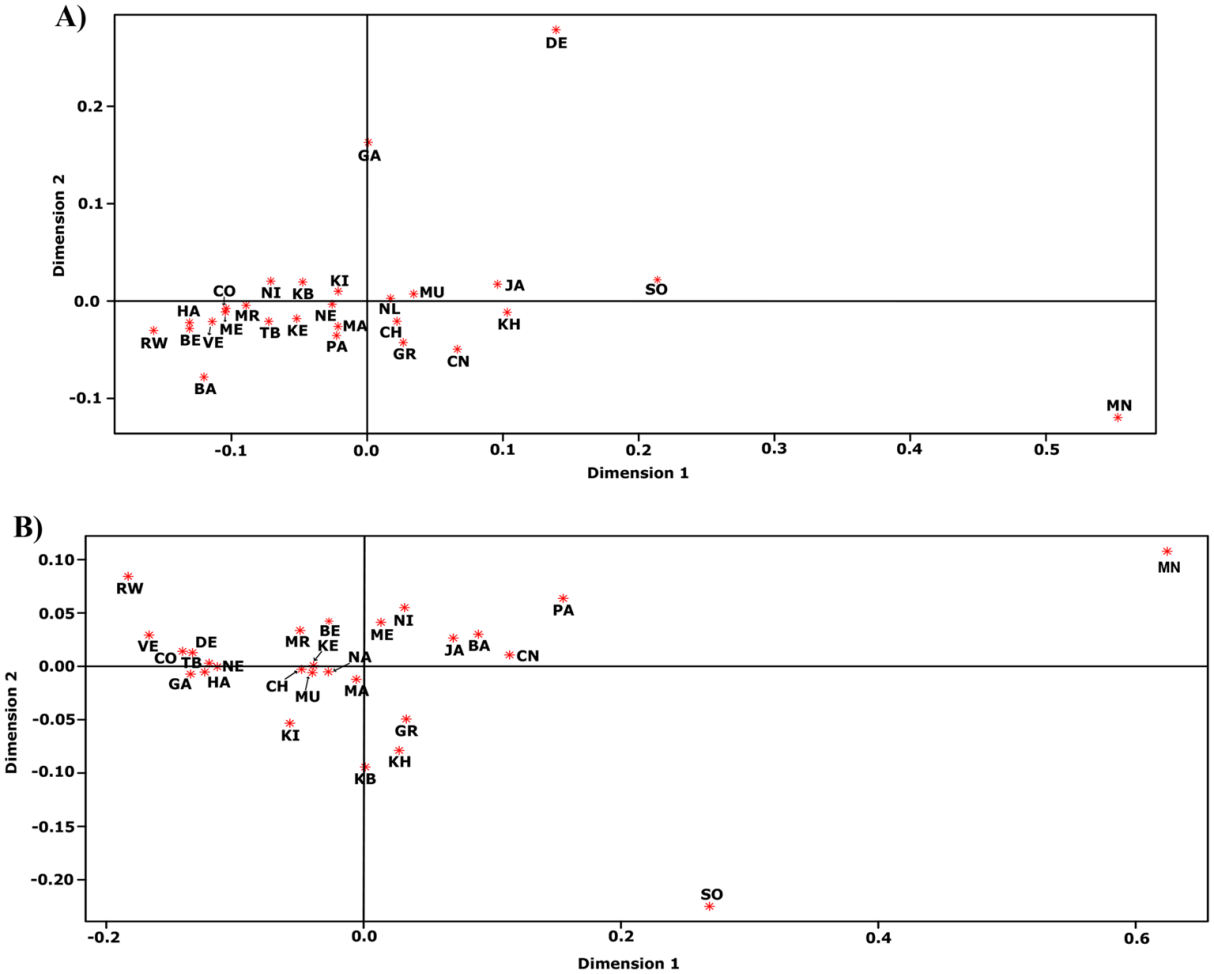
Multidimensional scaling plot (MDS) of Indian sheep breeds. The MDS plot was constructed using the pairwise *F*_ST_ values calculated for 746 D-loop (612 bp; (**A**)) and 738 *CYTB* gene (737 bp; (**B**)). *BE* Bellary, *CO* Coimbatore, *HA* Hassan, *KE* Kenguri, *KI* Kilakarsal, *MN* Mandya, *MR* Madras Red, *KB* Katchaikatty Black, *ME* Mecheri, *NI* Nilgiri, *RW* Ramnad White, *TB* Tiruchy Black, *VE* Vembur, *CN* Chottanagpuri, *BA* Balangir, *GA* Ganjam, *MU* Muzzafarnagri, *NL* Nali, *CH* Chokla, *KH* Kheri, *MA* Marwari, *JA* Jaisalmeri, *SO* Sonadi, *PA* Patanwadi, *DE* Deccani, *NE* Nellore, and *GR* Garole. The MDS plot was generated using R program (www.r-project.org) and the final output was edited in Inkscape 1.0 (https://inkscape.org).

**Figure 5:**
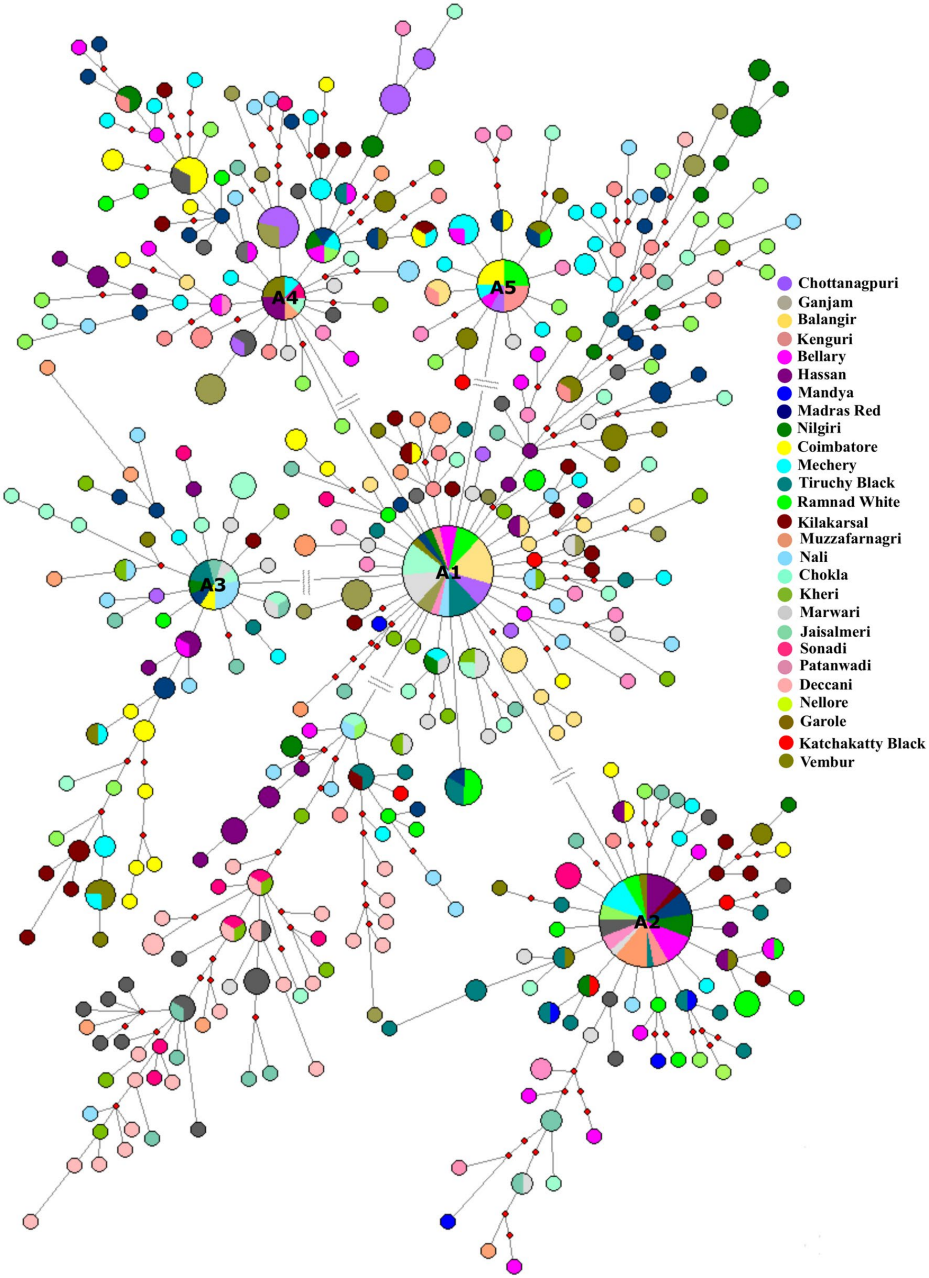
Median-joining network of Indian domestic sheep lineage A. The MJ network was constructed using 655 D-loop sequences (612 bp) belonging to 27 Indian sheep breeds. Size of the node is proportional to the number of sheep present in the node. The length of the line is proportional to the number of mutations (# indicates that the length of the line is not proportional to the number of mutations. There is only one mutation between the two haplotypes but length of the line has been adjusted for the convenient arrangement of the node). Sheep breeds are indicated by different colours. The small red rectangles represent the median vector. The network was constructed using Network 10.2.0.0^17^ and edited in Inkscape 1.0 (https://inkscape.org).

**Figure 6:**
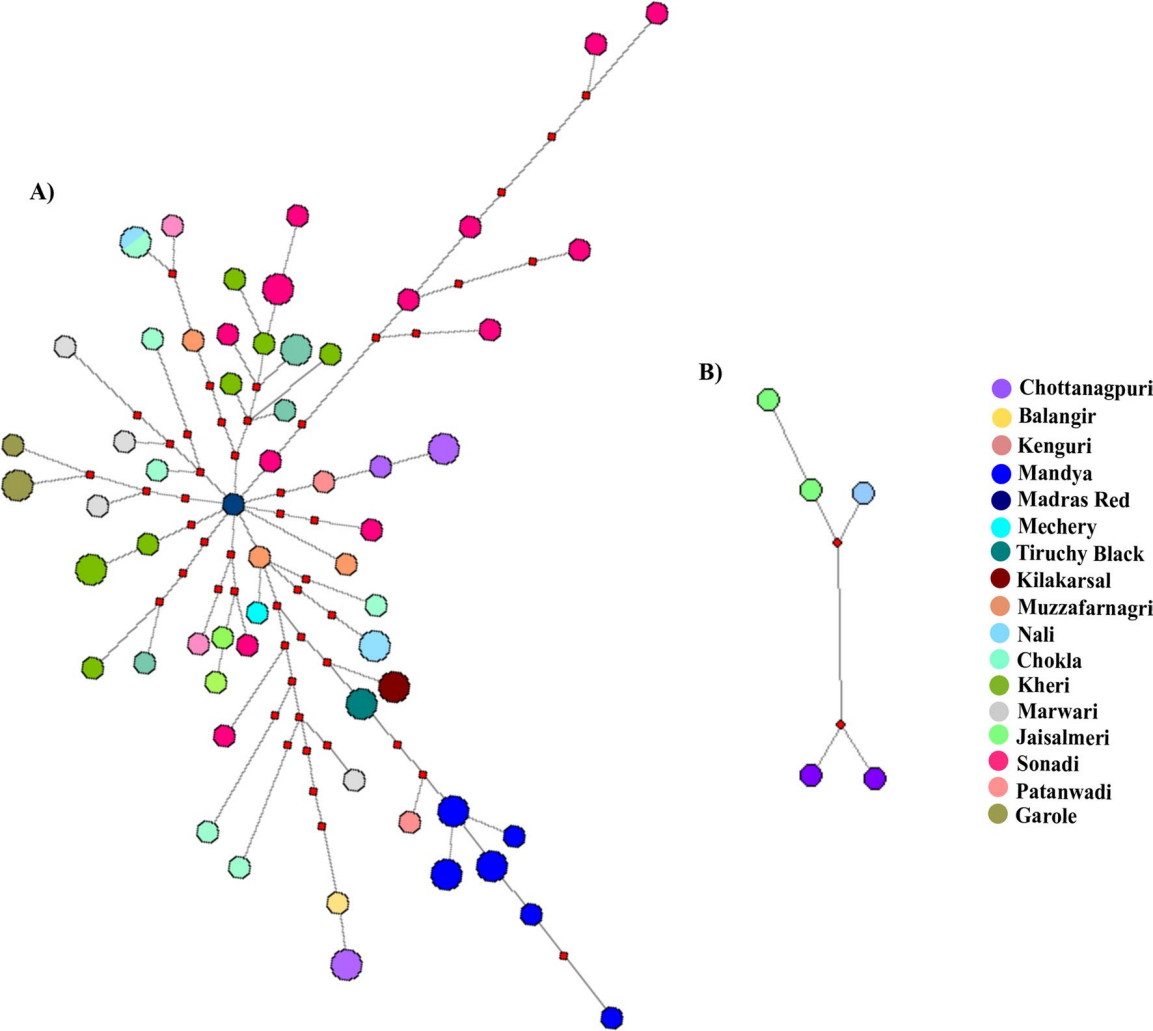
Median-joining network of Indian domestic sheep Lineage B and C. (**A**) The MJ network of lineage B was constructed using 612 bp D-loop sequences. A total of 86 sequences belonging to 17 domestic sheep breeds were used. (**B**) The MJ network of lineage C was constructed using five D-loop (612 bp) sequences belonging to three sheep breeds. The sizes of nodes are proportional to the number of sheep present in the node. The length of the line is proportional to the number of mutations. Sheep breeds are indicated by different colours. The small red rectangles represent the median vector. The network was constructed using Network 10.2.0.0^17^ and edited in Inkscape 1.0 (https://inkscape.org).

**Figure 7:**
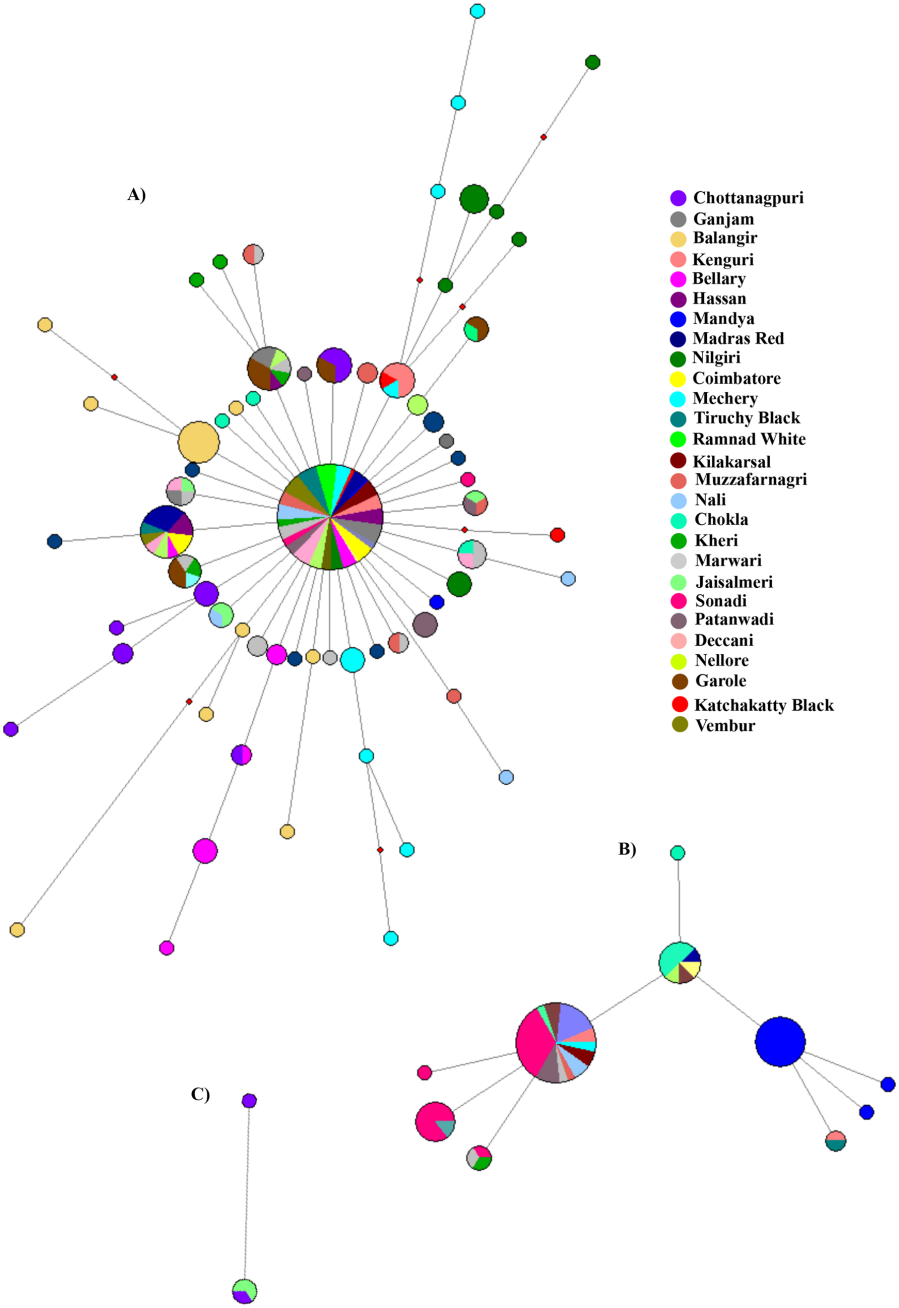
Median-joining network of Indian domestic sheep lineage A, B and C of *CYTB* gene sequences. (**A**) The MJ network of lineage A was constructed using 667 *CYTB* gene sequences (737 bp) of 27 Indian sheep breeds. (**B**) The MJ network of lineage B was constructed using 67 *CYTB* gene sequences (737 bp) of 17 sheep breeds. (**C**) The MJ network of lineage C was constructed using 4 *CYTB* gene (737 bp) sequences of two sheep breeds. Size of the node is proportional to the number of sheep present in the node. The length of the line is proportional to the number of mutations. Sheep breeds are indicated by different colours. The small red rectangles represent the median vector. The network was constructed using Network 10.2.0.0^17^ and edited in Inkscape 1.0 (https://inkscape.org).

To have a comprehensive view on the genetic diversity and phylogeography of Indian sheep breeds, we also sequenced three breeds (Chottanagpuri, Balangir and Ganjam), from north and east India, retrieved the mtDNA sequences of 12 Indian sheep breeds from GenBank and analysed them along with all new sequences from this study, and thus we included mtDNA sequences of 27 Indian sheep breeds in our analyses (746 D-loop and 738 *CYTB* gene sequences). The AMOVA showed 17.27% and 14.11% of variations among Indian breeds for D-loop and *CYTB* gene, respectively. However, when AMOVA was reperformed by excluding Sonadi and Mandya breeds, which were found to be distinct from others in the MDS plots (Fig. 4) the variations among the breeds were dropped down to 10.28% and 6.29% for D-loop and *CYTB* gene, respectively. Further MDS drawn using only lineage A sequences confirmed Sonadi and Mandya breeds to be distinct from other Indian sheep breeds due to the higher contribution of lineage B in these two breeds (Supplementary Fig. S5). Further, these 27 breeds were divided into four groups based on the agro-ecological regions of India for AMOVA to examine the effect of geographical locations on breed structuring. But this classification was not supported by the variation between these regions (2.42%). MJ networks were constructed separately for lineages of A, B and C using D-loop (Figs. 5, 6) and *CYTB* gene (Fig. 7) sequences to unveil the distribution patterns of different haplotypes in Indian sheep. There was no significant difference between south Indian and Indian (includes all Indian sequences) networks of lineages A and B while lineage C was only present in five animals belonging to Chottanagpuri (two), Jaisalmeri (two) and Nali (one) breeds (Fig. 6).

### Origin and migration of lineage A and B

The ABC analysis was capable to reconstruct the most probable colonization scenarios for lineages of A and B of the domestic sheep using D-loop sequences (Table 2). For lineage A, the scenario with the highest posterior probability (0.9500; 95% CI 0.9375–0.9625) identified the Indian subcontinent as the source population, which first spread to the Middle East region and then expand to the Mongolian Plateau. It indicated that the domestication of sheep occurred in Indian subcontinent much earlier than previously reported. The posterior predictive error was considerably low (0.285). The posterior distributions of demographic parameters were inferred under the best colonization scenario (Supplementary Table S3). Most parameter estimates showed high RMAE values (> 0.2) and cannot be considered fully reliable. However, because our main goal was to understand the origin of domestic sheep mtDNA lineages and colonization routes, the high RMAE values did not affect our evaluation of the drawn and tested scenarios. For lineage B, ABC analysis identified the Arabian sea route as the most probable colonization route to the Indian subcontinent (posterior probability: 0.6801; 95% CI: 0.6442–0.7159). This scenario also showed low posterior predictive error (0.254), which represented high confidence in the chosen scenario. All estimated parameters of the best scenario and their RMAE can be also found in Supplementary Table S3.

**Table 2:** Bayesian posterior probabilities for each tested scenario using DIYABC v2.1 for lineages of A and B of domestic sheep.

Tested scenarios	Posterior probability (95% CI)	Winner scenario
**Lineage A**		
Scenario 1_ME_: The source population is in the ME. The lineage A spread first from ME to MP at time t1 and posteriorly from MP to IS at time t2	0.0500 (0.0375 – 0.0625)	
**Scenario 4**_**IS**_**: The source population is in the IS. Lineage A spread first from IS to ME at time t1 and then to MP at time t2**	**0.9500 (0.9375 – 0.9625)**	
**Lineage B**		
Scenario 1: Lineage B spread from ME to MP at time t1 and posteriorly from MP to IS at time t2 (Inland route)	0.3199 (0.2841 – 0.2558)	
**Scenario 2: The IS lineage B was originated from ME at t4 in an independent colonization event (Arabian sea route)**	**0.6801 (0.6442 – 0.7159)**	

For each lineage, the winner scenario has the highest probability highlighted in bold. Only the winner scenario for each lineage was graphically represented. Colours represent the populations: ME (the Middle East, red), MP (the Mongolian Plateau, blue) and IS (the Indian subcontinent, green).

## Discussion

Sheep domestication and phylogeography have been subject of long stretched discussion worldwide during the past a few decades. To have a comprehensive view on the origin and phylogeography of Indian sheep breeds, we analysed the mtDNA D-loop and *CYTB* gene sequences of 27 Indian sheep breeds. The lineage A was predominant whereas lineage B was observed at a low frequency among Indian sheep as was the scenario in Chinese sheep breeds^22–26^. Surprisingly, 79% of samples of Mandya breed from south India were of lineage B. It may be recalled that Sonadi breed from northwestern India also had relatively high frequency (48%) of lineage B^8^ (Fig. 1). Lv et al.^11^ suggested that the lineage B entered into India through Mongolian Plateau. In such a scenario, one would expect a gradient of lineage B from north to south India. But the high frequencies of lineage B in Mandya breed from south India and in Sonadi breed from northwestern India would argue against the proposal of Lv et al.^11^. It may also be noted that the native tract of these two breeds are close to the Arabian Sea. The ABC analysis strongly supported the migration of lineage B type sheep from the Middle East into the Indian subcontinent through Arabian Sea route. Therefore, we support the earlier suggestion of Singh et al.^8^ that the lineage B might have arrived into India through sea route rather from the Mongolian Plateau as argued by Lv et al.^11^. The sea route has been used to export elephant and buffalo from India to other countries in the ancient times^27,28^ and this further strengthens our findings. Lineage C was only observed among sheep breeds of north and east India at a very low frequency (< 1%) due probably to its recent introduction into the Indian subcontinent as mentioned in the previous study^11^.

**Figure 8:**
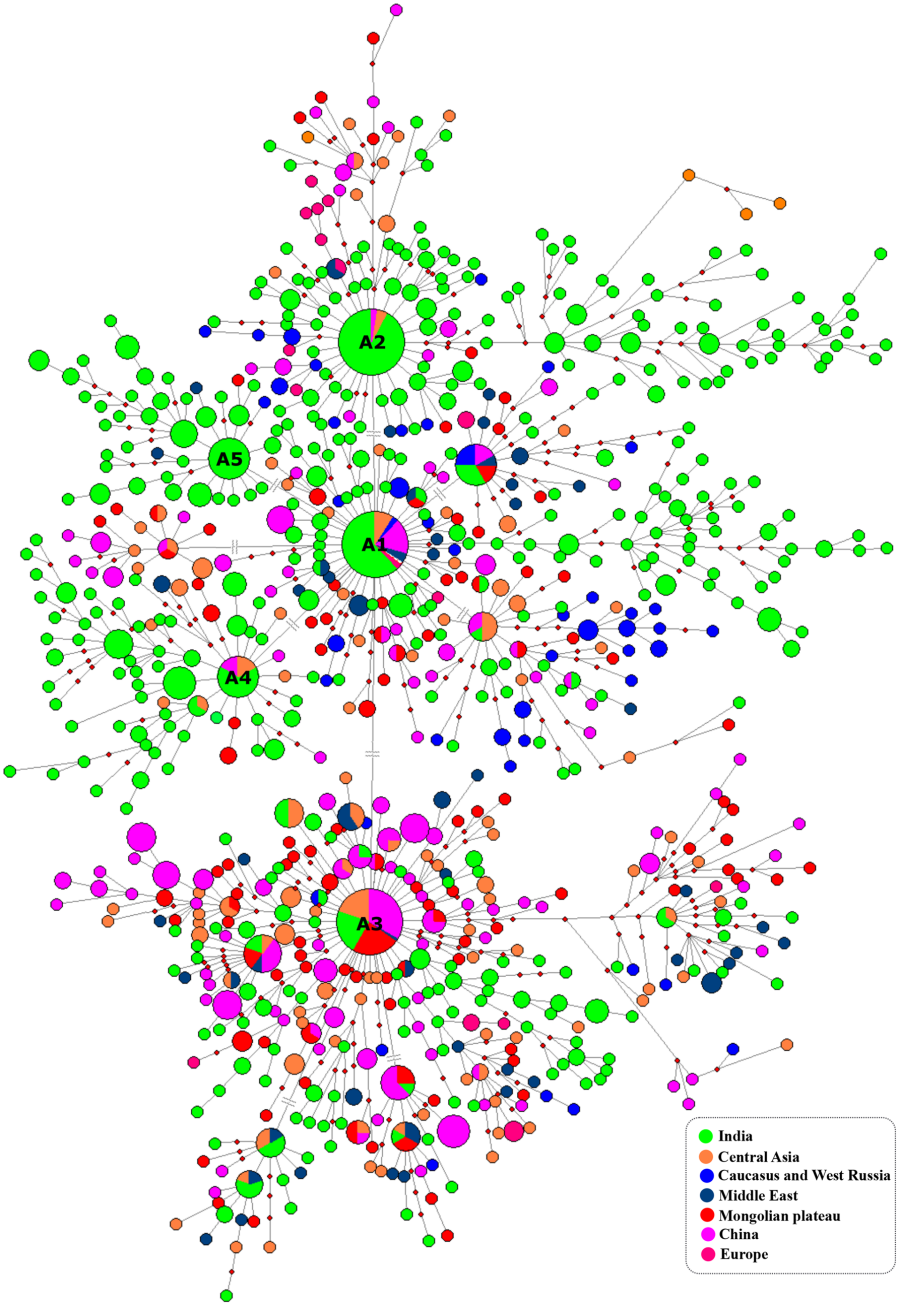
Median-joining network of domestic sheep lineage A. MJ network was constructed using 1303 D-loop sequences (482 bp) of domestic sheep belonging to India, China, Central Asia, Caucasus and West Russia, Middle East, Mongolia and Europe. GenBank accession numbers of the domestic sheep sequences are provided in the Supplementary Table S1. The sizes of nodes are proportional to the number of sheep present in the node. The length of the line is proportional to the number of mutations (# indicates that the length of the line is not proportional to the number of mutations. There is only one mutation between the two haplotypes, but length of the line has been adjusted for the convenient arrangement of the node). The colour of the node indicates the sampling location of sheep. The small red rectangles represent the median vector. The network was constructed using Network 10.2.0.0^17^ and edited in Inkscape 1.0 (https://inkscape.org).

Observing the immense diversity of lineage A in Indian sheep breeds, we constructed a MJ network for lineage A using D-loop sequences of Indian sheep and sheep from other regions of the world to understand the diversity and relationship of lineage A haplotypes. The MJ network was extremely complex and the haplotype diversity was higher in Indian sheep as compared to that of sheep breeds of other regions (Fig. 8). Only in Indian sheep, all the five major haplogroups of A1, A2, A3, A4 and A5 were found in contrast to lineage A sheep from any other country. Specifically, the Indian sheep had higher frequency of haplogroups of A2, A4 and A5. In addition, there were several singleton haplotypes in Indian sheep. The presence of multiple expanding haplogroups in Indian sheep breeds suggested a population expansion of lineage A in India. The same was also reflected in the mismatch distribution curve (Supplementary Fig. S6).

**Figure 9:**
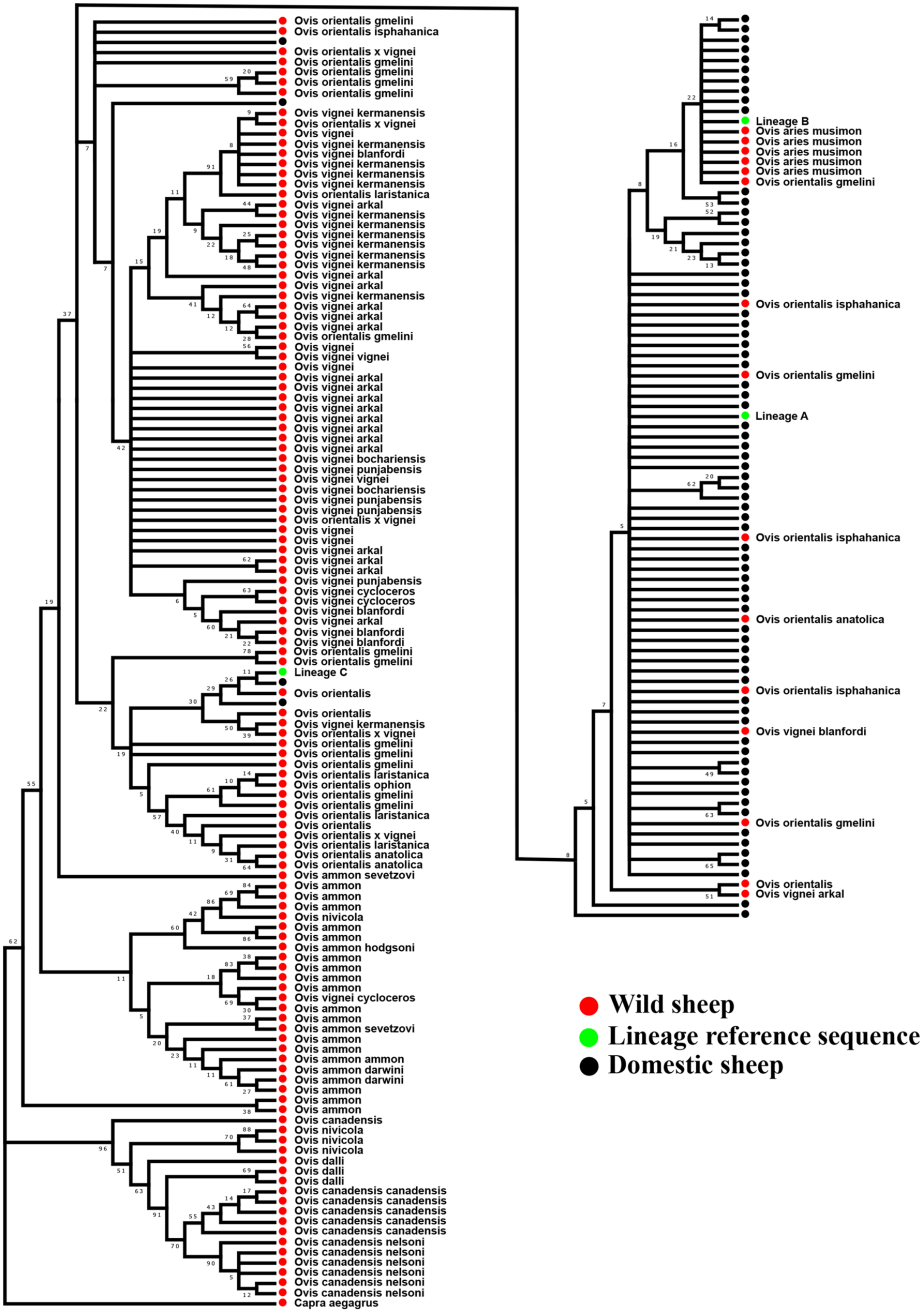
Neighbour-joining tree of Indian domestic sheep and wild sheep. The NJ tree was constructed using 737 bp *CYTB* gene sequence of domestic and wild sheep. The bootstrap values are given above/below the branches. GenBank accession numbers of the wild sheep sequences are provided in the Supplementary Table S1. The NJ tree was constructed using the software MEGA (version 7)^14^ and the final output was edited in Inkscape 1.0 (https://inkscape.org).

The wild sheep *Ovis orientalis* (*O. gmelini* as per recent nomenclature) is comprised of a number of subspecies which are mostly distributed in the Middle East and Southwest Asia, including the Indian subcontinent^29^. It has been suggested to be the wild ancestor of the modern-day domestic sheep^3,11,30,31^. Demirci et al.^31^ suggested that, the present-day domestic sheep might have originated from two maternally distinct ancestral *O. gmelini* populations. Lv et al.^11^ observed the close genetic relationship of *O. musimon* and *O. orientalis* with domestic sheep lineage B based on the whole mitogenome. However, the D-loop and *CYTB* gene based phylogenies were different from the whole mitogenome wherein the *O. musimon* and *O. orientalis* clustered with lineages A, B, and C of domestic sheep. This leaves open copious questions corresponding to the wild ancestors and their contribution to the gene pool of modern domestic sheep. Therefore, we constructed NJ trees using mtDNA D-loop and *CYTB* gene sequences of Indian domestic sheep and various wild sheep in order to identify the wild ancestor of Indian domestic sheep. The NJ tree of D-loop showed that Indian domestic sheep lineage A shared ancestry with wild species *O. orientalis anatolica* whereas lineage B shared ancestry with *O. musimon* and *O. Orientalis* (Supplementary Fig. S7) which is in accordance with previous studies^11^. In case of *CYTB* gene, the *O. orientalis* subspecies (*O. orientalis gmelini*, *O. orientalis anatolica* and *O. orientalis isphahanica*) clustered with lineage A while *O. aries musimon* and *O. orientalis gmelini* clustered with lineage B as has been reported previously^11^. We found that one of the wild *O. vignei blanfordi* sample (collected from Pakistan) clustered with lineage A of Indian domestic sheep (Fig. 9). Its close phylogenetic relationship with lineage A raises speculations that *O. vignei blanfordi* could be a possible wild ancestor of the lineage A of Indian domestic sheep. The distribution of *O. vignei blanfordi* in the Balochistan and Sindh (Pakistan) helps further in strengthening the argument that lineage A might have been domesticated in the Indian subcontinent.

Chen et al.^22^ have argued that sheep might have been domesticated independently in multiple places and not only confined to the Near East as proposed elsewhere. Similarly, Singh et al.^8^ proposed that the Indian subcontinent as the one of domestication centres for the lineage A sheep. The Indus valley region of the Indian subcontinent is regarded to be one of the earliest centres of domestication for several plant and animal species^32–35^. The presence of wild cattle, wild goat and wild sheep has been reported in Baluchistan in the sixth millennium B.C. through archaeological evidences^36^. The horncores of wild sheep have also been excavated from Mehrgarh (Neolithic period) and Nausharo (Harapan period)^37,38^. Further, sheep and goat remains have been recovered from the Central India (Indo-Gangetic Plain and Belan river valley) dated to 12,000–8000 BC^39^. Since animal domestication takes place locally, Jarrige and Meadow^36^ opined that inhabitant of Mehrgarh would have domesticated the sheep. The higher genetic diversity of Indian domestic sheep breeds gives strength to this proposition as it is expected that the domestication centres would have higher genetic variability^8,40,41^. Further, the ABC analysis also provides strength to the argument of Indian subcontinent as one of the domestication centres for lineage A by predicting independent origin of Indian sheep from the Middle East sheep. Therefore, based on our findings, previous molecular studies and archaeological evidences, we suggest that lineage A might have been domesticated in the Indian subcontinent.

In conclusion, our study suggests Indian subcontinent as one of the domestication centres for the lineage A sheep. The study also supports the earlier conclusion that the lineage B might have entered into India through sea route. Further investigation on *O. vignei blanfordi* from Pakistan is likely to extend understanding of the domestication history of sheep in Indian subcontinent.

## Supplementary Information

Supplementary Information.
