# Supplementary material for: Evidence for independent domestication of sheep mtDNA lineage A in India and introduction of lineage B through Arabian sea route
Source: Sci Rep. 2021 Oct 5;11:19733. doi: 10.1038/s41598-021-97761-y (PMC8492717; doi:10.1038/s41598-021-97761-y)
Supplement: Supplementary file 1 — Supplementary Information. [file 41598_2021_97761_MOESM1_ESM.pdf]

Evidence for independent domestication of sheep mtDNA lineage A in India and introduction of lineage B through Arabian Sea route

Ranganathan Kamalakkannan[1] (https://orcid.org/0000-0001-8378-4629), Satish Kumar[2], Karippadakam Bhavana[1] (https://orcid.org/0000-0002-8287-9957), Vandana R. Prabhu[1] (https://orcid.org/0000-0002-6302-8022), Carolina Barros Machado[3] (https://orcid.org/0000-0001-5195-5577), Hijam Surachandra Singha[1] (https://orcid.org/0000-0002-3108-0846), Dhandapani Sureshgopi[1] (https://orcid.org/0000-0001-9128-9572), Vincy Vijay[1], and Muniyandi Nagarajan[1*] (https://orcid.org/0000-0001-8328-9767)

[1]Department of Genomic Science

School of Biological Sciences

Central University of Kerala

Kasaragod-671316, Kerala, India

[2]Department of Biotechnology

School of Interdisciplinary and Applied Sciences

Central University of Haryana

Mahendergargh-123029, Haryana, India

[3]Department of Genetic and Evolution

Federal University of São Carlos

Rodovia Washington Luís, Km235 - SP-310, São Paulo, Brazil

* Corresponding author email: nagarajan@cukerala.ac.in

Supplementary Figure S1. First round of colonization scenarios tested using DIYABC in lineage A of domestic sheep. We built two independent models [(A) and (B)] with four scenarios each one, considering as the main difference between them the source population identity: (A) Middle East is the source population and (B) Indian is the source population. Colours represent the populations: ME (Middle East, red), MP (Mongolian Plateau, blue) and IS (Indian subcontinent, green). The scenarios with highest posterior probabilities (PP) are in bold and was tested against each other in the second round.

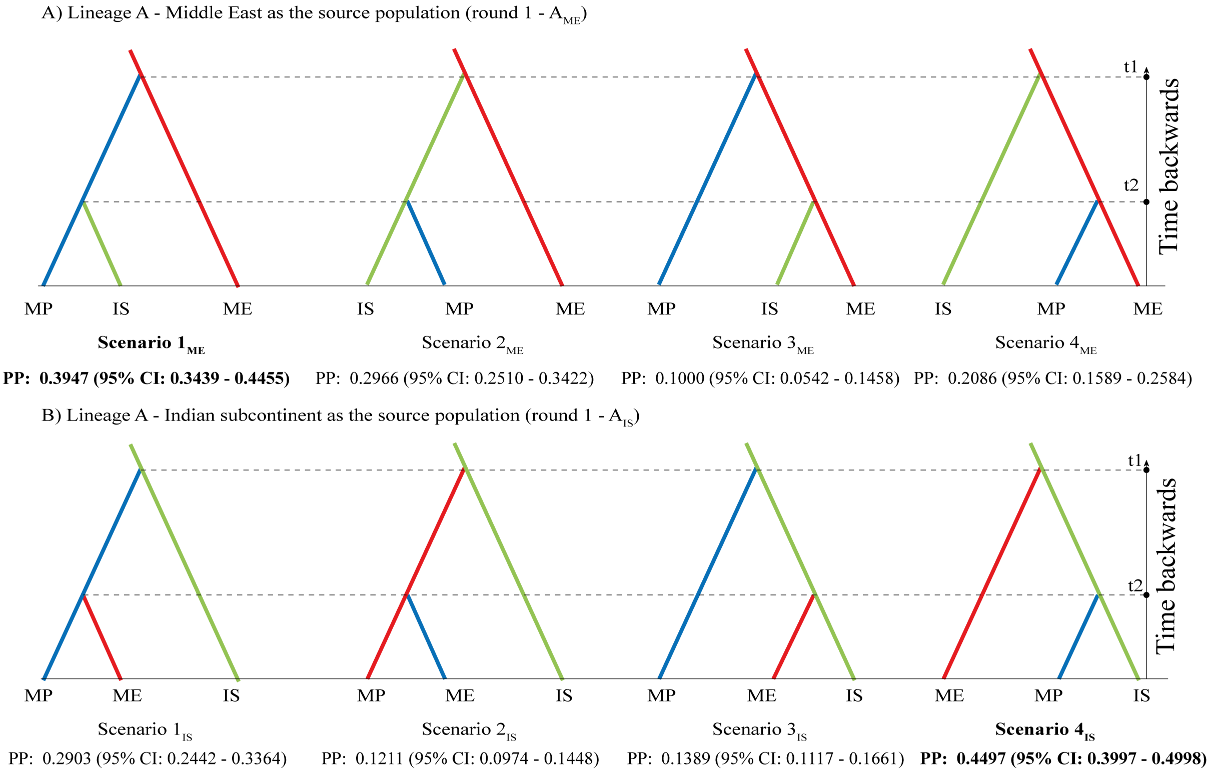

Supplementary Figure S2. Multidimensional scaling plot of south Indian domestic sheep. The MDS plot was generated using R program (www.r-project.org) and the final output was edited in Inkscape 1.0 (https://inkscape.org).

A) D-loop

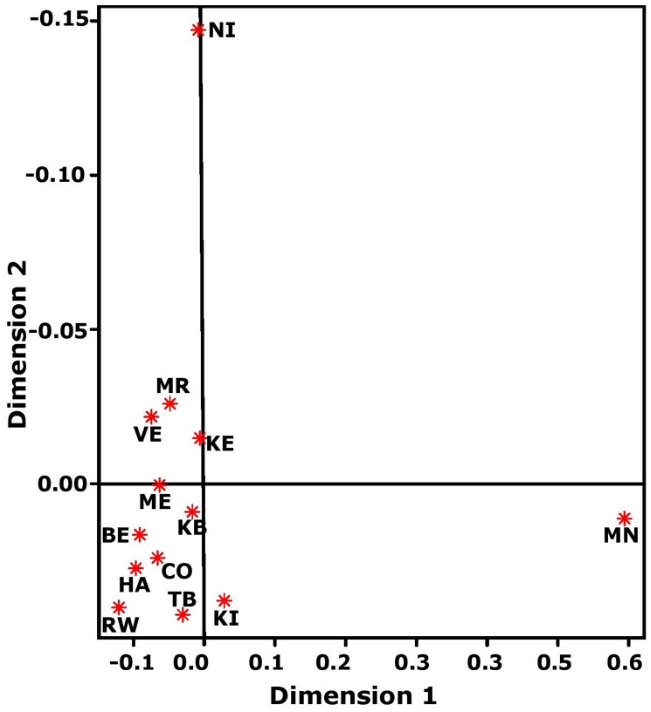

B) *CYTB* gene

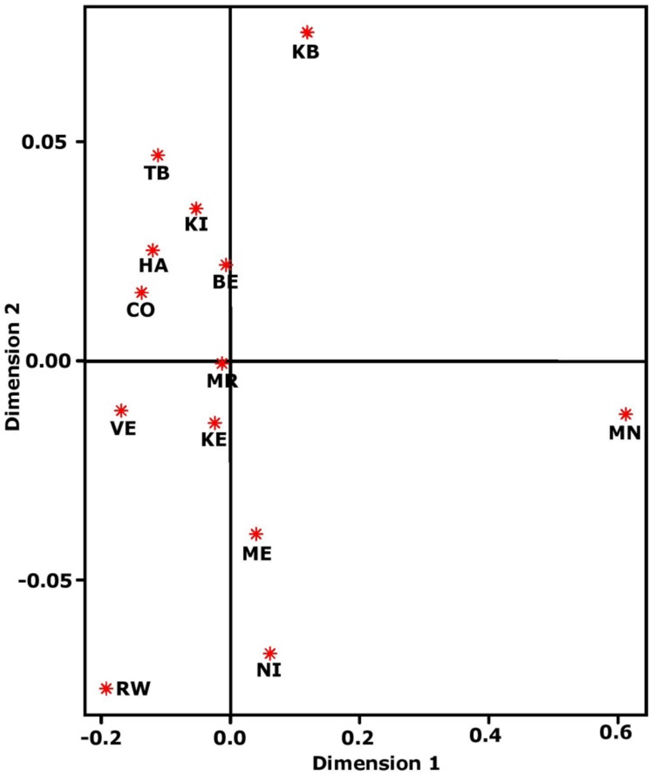

Supplementary Figure S3. Median joining network of South Indian domestic sheep. The MJ network was constructed using 950 bp D-loop sequences. A total of 359 sequences belonging to 13 domestic sheep breeds were used. The sizes of nodes are proportional to the number of animals present in the node. The length of the line is proportional to the number of mutations. In certain cases (#) the length of the line is not proportional to the number of mutations (there is only one mutation between the two haplotypes) but length of the line has been adjusted for the convenient arrangement of the node. Sheep breeds are indicated by different colours. The small red rectangles represent the median vector. A: Lineage A; B: Lineage B. The network was constructed using Network 10.2.0.0[17] and edited in Inkscape 1.0 (https://inkscape.org).

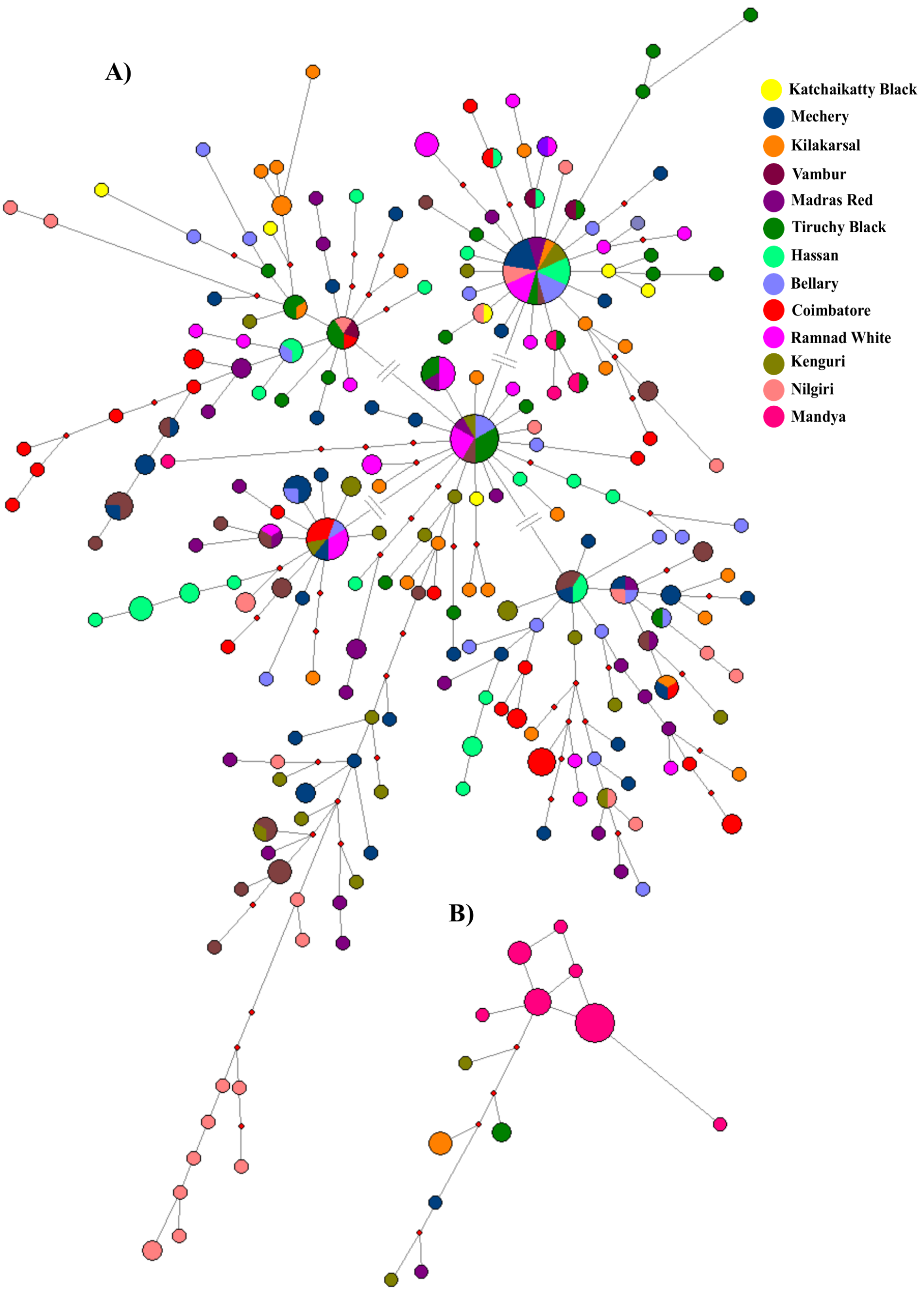

Supplementary Figure S4. Median joining network of South Indian domestic sheep. The MJ network was constructed using 856 bp *CYTB* gene sequences. A total of 352 sequences belonging to 13 domestic sheep breeds were used. The sizes of nodes are proportional to the number of sheep present in the node. The length of the line is proportional to the number of mutations. Sheep breeds are indicated by different colours. The small red rectangles represent the median vector. A: Lineage A; B: Lineage B. The network was constructed using Network 10.2.0.0[17] and edited in Inkscape 1.0 (https://inkscape.org).

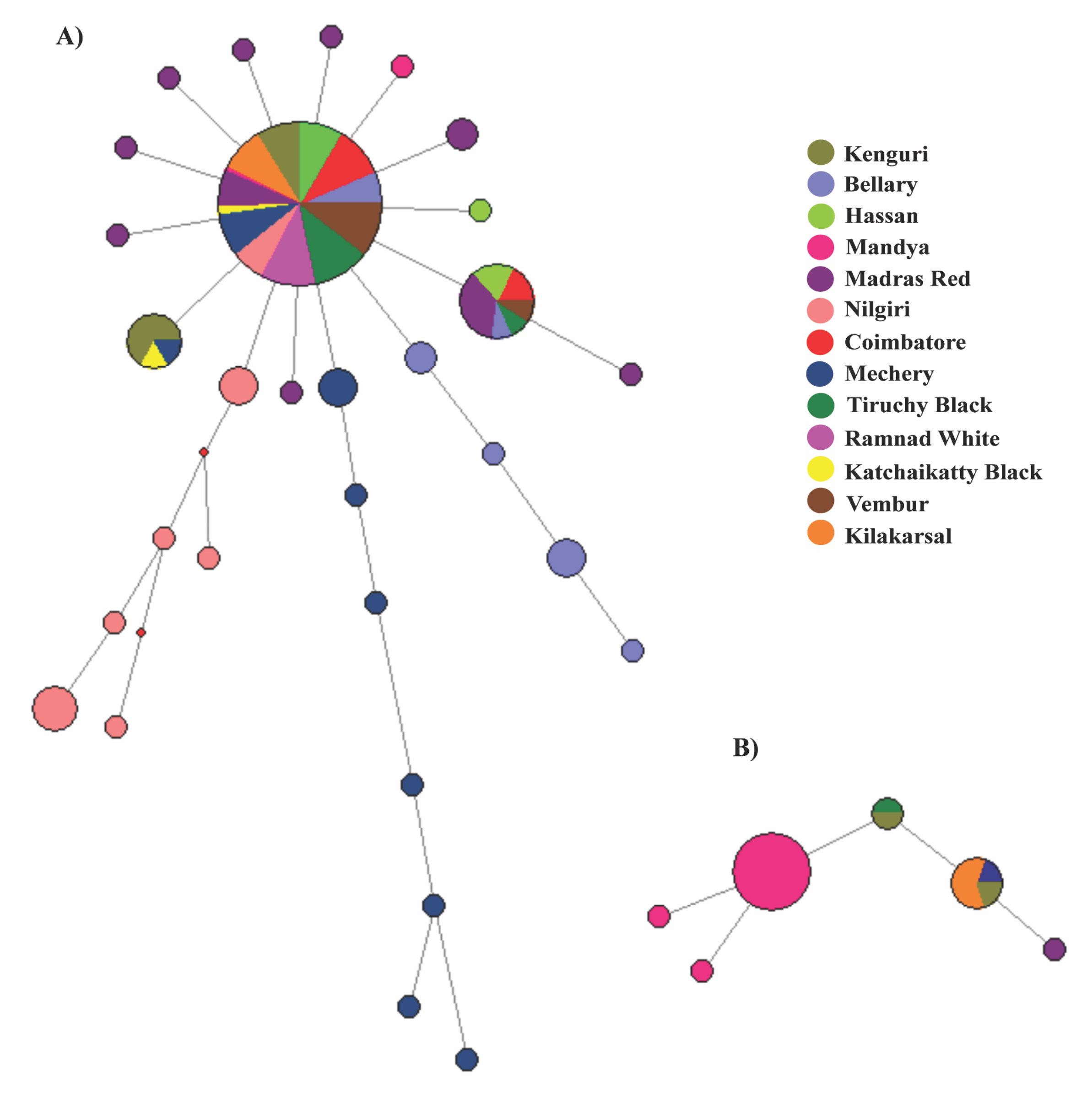

Supplementary Figure S5. Multidimensional scaling plot of Indian domestic sheep Lineage A. A: D-loop; B: *CYTB* gene. The MDS plot was generated using R program (www.r-project.org) and the final output was edited in Inkscape 1.0 (https://inkscape.org).

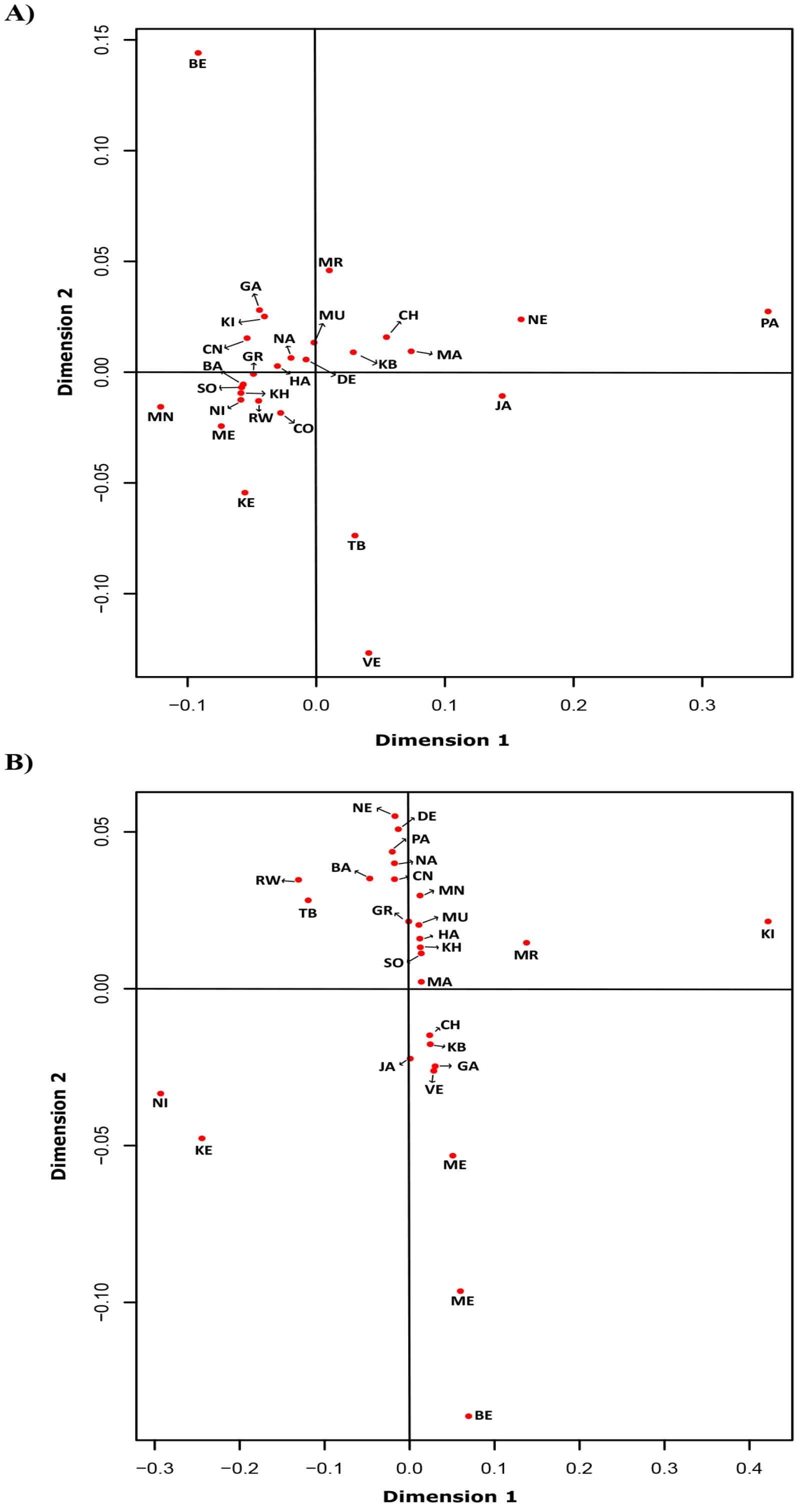

Supplementary Figure S6. Mismatch distribution curve of lineage A. Mismatch distribution curve was obtained using 612 bp D-loop sequence of 655 sheep samples. The observed mismatch distribution (green) was compared with the expected mismatch distribution (red) under sudden expansion model. The number of nucleotide differences between a pair of sequences is given in the *x* axis, and the respective frequency (%) is given in the *y* axis.

Lineage A

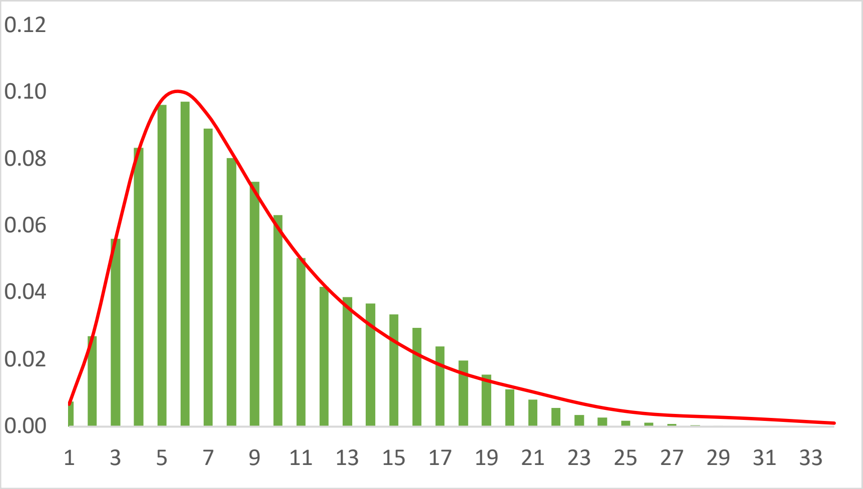

Supplementary Figure S7. Neighbour-joining tree of Indian domestic sheep and wild sheep. The NJ tree was constructed using 612 bp D-loop sequence of wild and domestic sheep. The bootstrap values were given above/below the branches. GenBank accession numbers of the wild sheep sequences are provided in the Supplementary Table S1. The NJ tree was constructed using the software MEGA (version 7)[14] and the final output was edited in Inkscape 1.0 (https://inkscape.org).

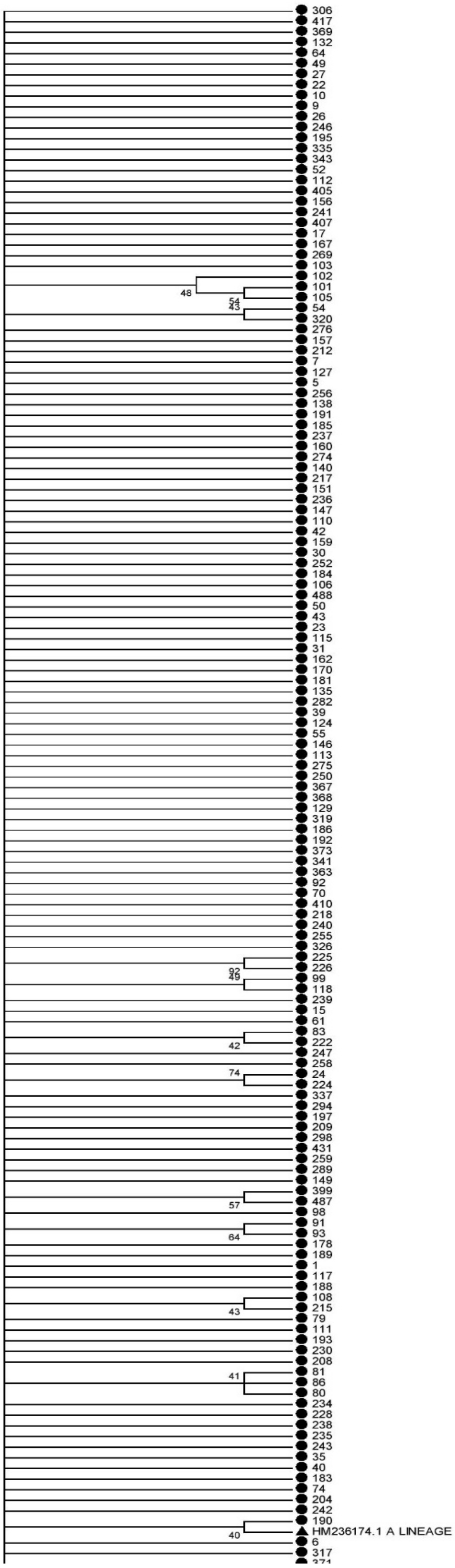

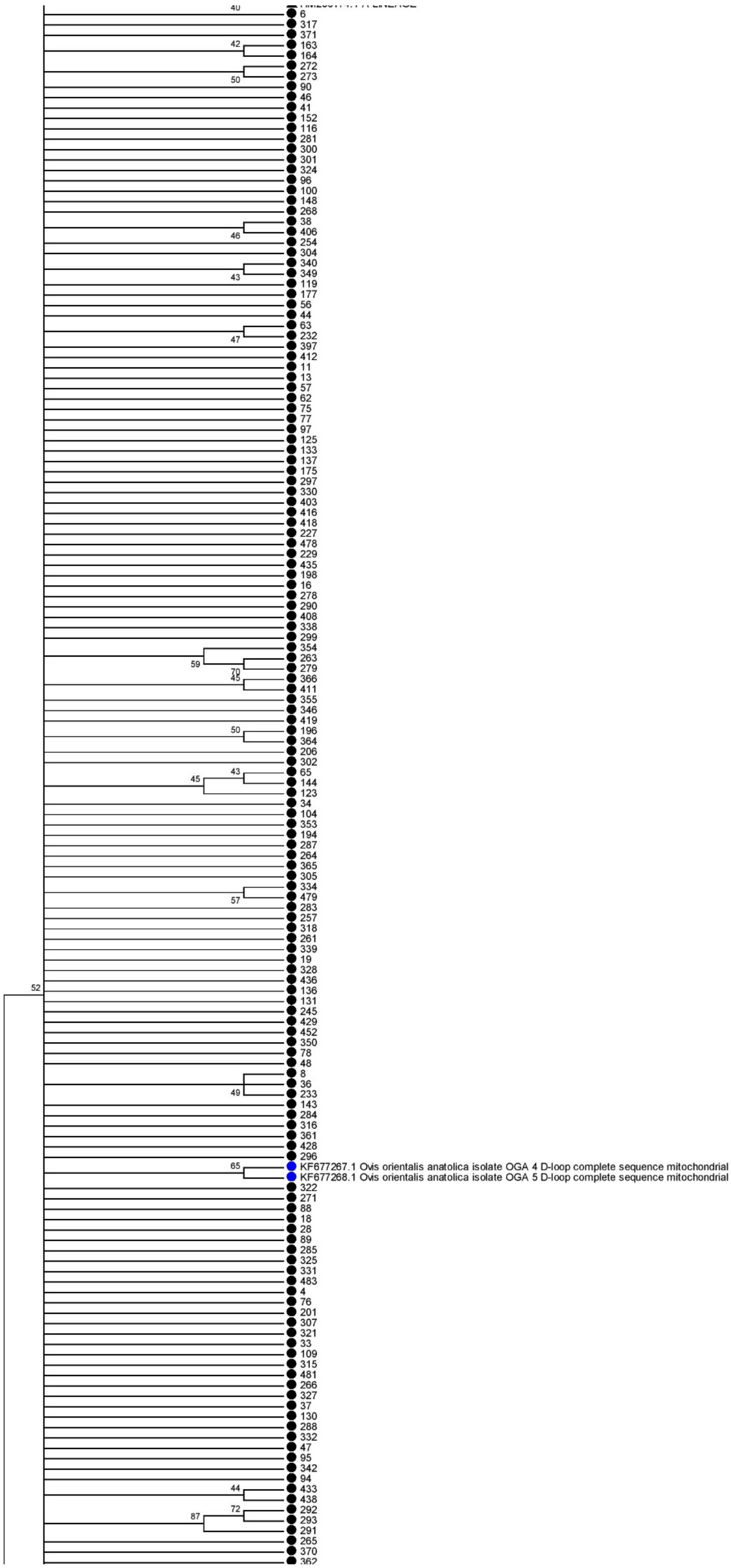

40
6
317
371
42
163
164
272
273
50
90
46
41
152
116
281
300
301
324
96
100
148
268
38
406
46
254
304
340
349
43
119
177
56
44
63
232
47
397
412
11
13
57
62
75
77
97
125
133
137
175
297
330
403
416
418
227
478
229
435
198
16
278
290
408
338
299
354
263
59
279
70
45
366
411
355
346
419
50
196
364
206
302
43
65
45
144
123
34
104
353
194
287
264
365
305
334
479
57
283
257
318
261
339
19
328
436
52
136
131
245
429
452
350
78
48
8
36
233
49
143
284
316
361
428
296
65
KF677267.1 Ovis orientalis anatolica isolate OGA 4 D-loop complete sequence mitochondrial
KF677268.1 Ovis orientalis anatolica isolate OGA 5 D-loop complete sequence mitochondrial
322
271
88
18
28
89
285
325
331
483
4
76
201
307
321
33
109
315
481
266
327
37
130
288
332
47
95
342
94
44
433
438
72
292
87
293
291
265
370
362

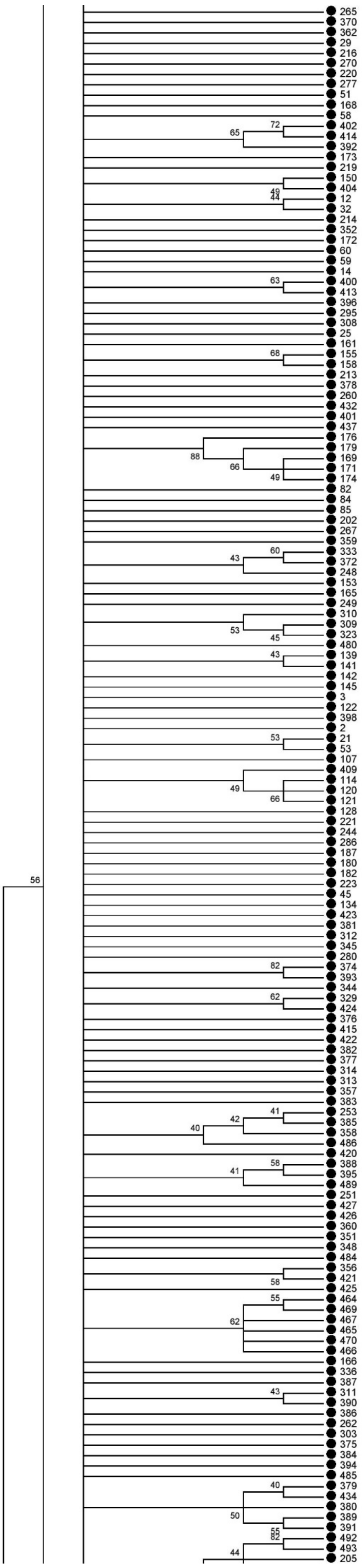
265
370
362
29
216
270
220
277
51
168
58
72
402
65
414
392
173
219
150
49
404
44
12
32
214
352
172
60
59
14
63
400
413
396
295
308
25
161
68
155
158
213
378
260
432
401
437
176
179
88
169
66
171
49
174
82
84
85
202
267
359
60
333
43
372
248
153
165
249
310
309
53
323
45
480
43
139
141
142
145
3
122
398
2
53
21
53
107
409
114
49
120
66
121
128
221
244
286
187
180
56
182
223
45
134
423
381
312
345
280
82
374
393
344
62
329
424
376
415
422
382
377
314
313
357
383
41
253
42
385
40
358
486
420
58
388
41
395
489
251
427
426
360
351
348
484
356
421
58
425
55
464
469
62
467
465
470
466
166
336
387
43
311
390
386
262
303
375
384
394
485
40
379
434
380
50
389
55
391
82
492
44
493
205

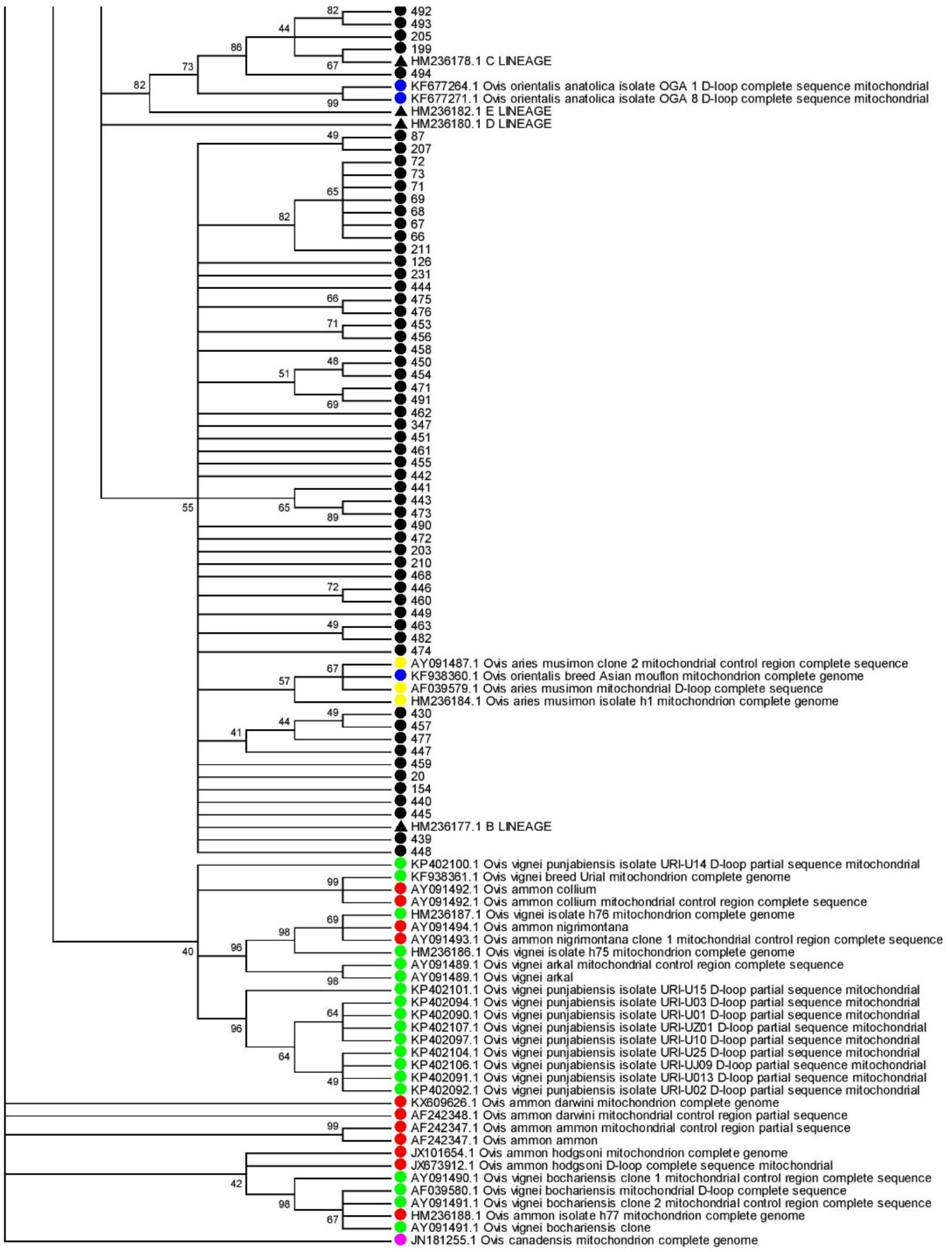
492
493
205
199
HM236178.1 C LINEAGE
494
KF677264.1 Ovis orientalis anatolica isolate OGA 1 D-loop complete sequence mitochondrial
KF677271.1 Ovis orientalis anatolica isolate OGA 8 D-loop complete sequence mitochondrial
HM236182.1 E LINEAGE
HM236180.1 D LINEAGE
87
207
72
73
71
69
68
67
66
211
126
231
444
475
476
453
456
458
450
454
471
491
462
347
451
461
455
442
441
443
473
490
472
203
210
468
446
460
449
463
482
474
AY091487.1 Ovis aries musimon clone 2 mitochondrial control region complete sequence
KF938360.1 Ovis orientalis breed Asian mouflon mitochondrion complete genome
AF039579.1 Ovis aries musimon mitochondrial D-loop complete sequence
HM236184.1 Ovis aries musimon isolate h1 mitochondrion complete genome
430
457
477
447
459
20
154
440
445
HM236177.1 B LINEAGE
439
448
KP402100.1 Ovis vignei punjabiensis isolate URI-U14 D-loop partial sequence mitochondrial
KF938361.1 Ovis vignei breed Urial mitochondrion complete genome
AY091492.1 Ovis ammon collium
AY091492.1 Ovis ammon collium mitochondrial control region complete sequence
HM236187.1 Ovis vignei isolate h76 mitochondrion complete genome
AY091494.1 Ovis ammon nigrimontana
AY091493.1 Ovis ammon nigrimontana clone 1 mitochondrial control region complete sequence
HM236186.1 Ovis vignei isolate h75 mitochondrion complete genome
AY091489.1 Ovis vignei arkal mitochondrial control region complete sequence
AY091489.1 Ovis vignei arkal
KP402101.1 Ovis vignei punjabiensis isolate URI-U15 D-loop partial sequence mitochondrial
KP402094.1 Ovis vignei punjabiensis isolate URI-U03 D-loop partial sequence mitochondrial
KP402090.1 Ovis vignei punjabiensis isolate URI-U01 D-loop partial sequence mitochondrial
KP402107.1 Ovis vignei punjabiensis isolate URI-UZ01 D-loop partial sequence mitochondrial
KP402097.1 Ovis vignei punjabiensis isolate URI-U10 D-loop partial sequence mitochondrial
KP402104.1 Ovis vignei punjabiensis isolate URI-U25 D-loop partial sequence mitochondrial
KP402106.1 Ovis vignei punjabiensis isolate URI-UJ09 D-loop partial sequence mitochondrial
KP402091.1 Ovis vignei punjabiensis isolate URI-U013 D-loop partial sequence mitochondrial
KP402092.1 Ovis vignei punjabiensis isolate URI-U02 D-loop partial sequence mitochondrial
KX609626.1 Ovis ammon darwini mitochondrion complete genome
AF242348.1 Ovis ammon darwini mitochondrial control region partial sequence
AF242347.1 Ovis ammon ammon mitochondrial control region partial sequence
AF242347.1 Ovis ammon ammon
JX101654.1 Ovis ammon hodgsoni mitochondrion complete genome
JX673912.1 Ovis ammon hodgsoni D-loop complete sequence mitochondrial
AY091490.1 Ovis vignei bochariensis clone 1 mitochondrial control region complete sequence
AF039580.1 Ovis vignei bochariensis mitochondrial D-loop complete sequence
AY091491.1 Ovis vignei bochariensis clone 2 mitochondrial control region complete sequence
HM236188.1 Ovis ammon isolate h77 mitochondrion complete genome
AY091491.1 Ovis vignei bochariensis clone
JN181255.1 Ovis canadensis mitochondrion complete genome

Supplementary Table S1. GenBank accession numbers of the sheep sequences used in the study.

D-loop (domestic sheep)

DQ242050 DQ242053 DQ242054 DQ242055 DQ242057 DQ242058 DQ242116 DQ242118 DQ242119 DQ242125 DQ242126 DQ242174 DQ242181 DQ242184 DQ242196 DQ242200 DQ242201 DQ242202 DQ242203 DQ242210 DQ242214 DQ242215 DQ242063 DQ242064 DQ242067 DQ242068 DQ242069 DQ242078 DQ242091 DQ242095 DQ242096 DQ242098 DQ242102 DQ242103 DQ242104 DQ242105 DQ242111 DQ242113 DQ242127 DQ242129 DQ242131 DQ242133 DQ242134 DQ242135 DQ242161 DQ242164 DQ242165 DQ242166 DQ242172 DQ242216 DQ242223 DQ242225 DQ242227 DQ242230 DQ242233 DQ242248 DQ242249 DQ242250 DQ242257 DQ242260 DQ242266 DQ242273 DQ242275 DQ242312 DQ242318 DQ242397 DQ242400 DQ242402 DQ242406 DQ242411 DQ242414 DQ242431 DQ242442 DQ242444 DQ242148 DQ242149 DQ242150 DQ242156 DQ242157 DQ242297 DQ242455 DQ491576 DQ491578 DQ491580 DQ491581 DQ491582 DQ491710 EU019144 EU019149 EU019150 EU019185 DQ242306 DQ242307 DQ242310 KF677026 KF677029 KF677030 KF677033 KF677035 KF677045 KF677050 KF677051 KF677058 KF677059 KF677060 KF677062 KF677063 KF677065 KF677069 KF677074 KF677077 KF677080 KF677089 KF677091 KF677092 KF677095 KF677100 KF677102 KF677106 KF677108 KF677112 KF677113 KF677119 KF677124 KF677125 KF677128 KF677133 KF677135 KF677140 KF677141 KF677145 KF677146 KF677152 KF677153 KF677154 KF677155 KF677157 KF677164 KF677177 KF677190 KF677192 KF677197 KF677203 KF677206 KF677212 KF677215 KF677216 KF677220 KF677221 KF677224 KF677225 KF677238 KF677239 KF677246 KF677247 KF677256 KF677258 KF677260 KF677262 KF677024 KF677031 KF677032 KF677036 KF677038 KF677039 KF677043 KF677048 KF677049 KF677061 KF677064 KF677066 KF677067 KF677068 KF677070 KF677072 KF677073 KF677078 KF677079 KF677081 KF677082 KF677088 KF677093 KF677096 KF677097 KF677098 KF677104 KF677109 KF677118 KF677120 KF677122 KF677123 KF677127 KF677129 KF677130 KF677131 KF677132 KF677136 KF677138 KF677139 KF677143 KF677147 KF677148 KF677149 KF677150 KF677156 KF677159 KF677160 KF677163 KF677165 KF677166 KF677168 KF677170 KF677171 KF677172 KF677173 KF677176 KF677178 KF677179 KF677180 KF677182 KF677183 KF677185 KF677188 KF677189 KF677193 KF677199 KF677204 KF677207 KF677211 KF677213 KF677214 KF677217 KF677222 KF677226 KF677227 KF677229 KF677230 KF677232 KF677233 KF677234 KF677236 KF677237 KF677242 KF677244 KF677254 KR697985 KR697986 KR697987 KR697988 KR697989 KR697990 KR697998 KR698003 KR698004 KR698007 KR698008 FJ545893 FJ545894 FJ545895 FJ545898 FJ545900 FJ545902 FJ545904 FJ545906 FJ545908 FJ545909 FJ545911 FJ545913 FJ545914 FJ545915 FJ545916 FJ545922 FJ545926 FJ545927 FJ545930 FJ545935 FJ545937 FJ545938 FJ545942 FJ545944 FJ545948 FJ545958 FJ545959 FJ545960 FJ545961 FJ545963 FJ545964 FJ545967 FJ545968 FJ545971 FJ545972 FJ545973 FJ545975 FJ545976 FJ545977 FJ545978 FJ545981 FJ545982 FJ545983 FJ545984 FJ545985 FJ545986 FJ545987 FJ545989 FJ545990 FJ545991 FJ545992 FJ545993 FJ545995 FJ545998 FJ546001 FJ546003 FJ546004 FJ546006 FJ546009 FJ546012 FJ546013 FJ546014 FJ546015

FJ546016 FJ546017 FJ546020 FJ546021 FJ546022 FJ546024 FJ546029 FJ546030 FJ546031 FJ546032 FJ546033 FJ546034 FJ546035 FJ546036 FJ546038 FJ546039 FJ546045 FJ546046 FJ546047 FJ546048 FJ546049 FJ546050 FJ546051 FJ546052 FJ546053 FJ546054 FJ546055 FJ546056 FJ545892 FJ545896 FJ546043 FJ545897 FJ545901 FJ545903 FJ546044 FJ545910 FJ545912 FJ545920 FJ545921 FJ545923 FJ545925 FJ545928 FJ545929 FJ545932 FJ545940 FJ545941 FJ545936 FJ545946 FJ545949 FJ545952 FJ545954 FJ545956 FJ546057 FJ545962 FJ545965 FJ545966 FJ545969 FJ545970 FJ545979 FJ545980 FJ545996 FJ545997 FJ546000 FJ545988 FJ546005 FJ546007 FJ546018 FJ546023 FJ546025 FJ546026 FJ546028 FJ546042 KP000283 KP000284 KP000285 KP000286 KP000288 KP000292 KP000293 KP000295 KP000296 KP000297 KP000298 KP000299 KP000300 KP000302 KP000304 KP000309 KP000313 KP000314 KP000319 KP000320 KP000321 KP000323 KP000325 KP000326 KP000331 KP000332 KP000333 KP000336 KP000337 KP000338 KP000339 KP000341 KP000343 KP000345 KP000346 KP000348 KP000351 KP000352 KP000353 KP000354 KP000358 KP000359 KP000355 KP000357 KP000360 KP000362 KP000350 KP000347 KP000287 KP000289 KP000291 KP000301 KP000303 KP000312 KP000315 KP000316 KP000317 KP000318 KP000311 KP000328 KP000329 KP000330 KP000335 KP000342 KP000344 KP000340 KP000036 KP000037 KP000038 KP000039 KP000041 KP000042 KP000044 KP000046 KP000047 KP000048 KP000052 KP000058 KP000059 KP000060 KP000068 KP000070 KP000073 KP000074 KP000077 KP000079 KP000085 KP000087 KP000088 KP000089 KP000091 KP000092 KP000093 KP000094 KP000095 KP000096 KP000099 KP000100 KP000102 KP000103 KP000104 KP000106 KP000107 KP000108 KP000109 KP000110 KP000112 KP000113 KP000114 KP000116 KP000117 KP000121 KP000122 KP000125 KP000126 KP000127 KP000130 KP000132 KP000133 KP000134 KP000135 KP000136 KP000137 KP000140 KP000141 KP000142 KP000143 KP000147 KP000148 KP000150 KP000151 KP000154 KP000155 KP000158 KP000159 KP000161 KP000163 KP000164 KP000165 KP000167 KP000168 KP000170 KP000175 KP000176 KP000178 KP000179 KP000180 KP000181 KP000187 KP000188 KP000189 KP000191 KP000193 KP000199 KP000200 KP000201 KP000203 KP000204 KP000205 KP000208 KP000209 KP000211 KP000212 KP000213 KP000215 KP000216 KP000217 KP000218 KP000221 KP000223 KP000224 KP000226 KP000227 KP000228 KP000230 KP000231 KP000232 KP000233 KP000238 KP000240 KP000241 KP000242 KP000243 KP000245 KP000246 KP000249 KP000250 KP000251 KP000252 KP000254 KP000255 KP000256 KP000257 KP000258 KP000261 KP000263 KP000264 KP000266 KP000269 KP000270 KP000271 KP000273 KP000274 KP000276 KP000364 KP000366 KP000371 KP000373 KP000376 KP000381 KP000382 KP000383 KP000384 KP000388 KP000389 KP000390 KP000391 KP000392 KP000393 KP000394 KP000395 KP000397 KP000398 KP000399 KP000400 KP000402 KP000404 KP000405 KP000406 KP000407 KP000409 KP000411 KP000413 KP000414 KP000415 KP000416 KP000420 KP000421 KP000422 KP000423 KP000424 KP000425 KP000426 KP000436 KP000441 KP000453 KP000455 KP000456 KP000457 KP000459 KP000468 KP000469 KP000471 KP000473 KP000475 KP000476 KP000477 KP000478 KP000481 KP000482 KP000490 KP000499 KP000500 KP000501 KP000502 KP000519 KP000521 KP000524 KP000525 KP000526 KP000527 KP000537 KP000539 KP000540 KP000544 KP000546 KP000551 KP000552 KP000555 KP000557 KP000559 KP000563 KP000564 KP000565 KP000566 KP000567 KP000568 KP000569

KP000578 KP000581 KP000582 KP000584 KP000585 KP000588 KP000592 KP000593
KP000594 KP000595 KP000598 KP000599 KP000602 KP000604 KP000608 KP000613
KP000616 KP000617 KP000618 KP000619 KP000621 KP000622 KP000623 KP000625
KP000626 KP000627 KP000636 KP000644 KP000646 KP000648 KP000649 KP000650
KP000651 KP000653 KP000654 KP000655 KP000659 KP000664 KP000666 KP000668
KP000680 KP000681 KP000682 KP000683 KP000684 KP000687 KP000691 KP000692
KP000693 KP000694 KP000695 KP000697 KP000699 KP000702 KP000703 KP000705
KP000708 KP000713 KP000714 KP000717 KP000721 KP000724 KP000725 KP000728
KP000729 KP000730 KP000731 KP000734 KP000735 KP000736 KP000737 KP000739
KP000740 KP000742 KP000745 KP000748 KP000751 KP000752 KP000753 KP000754
KP000755 KP000758 KP000759 KP000760 KP000767 KP000768 KP000770 KP000775
KP000776 KP000779 KP000781 KP000782 KP000783 KP000784 KP000785 KP000786
KP000787 KP000788 KP000791 KP000793 KP000794 KP000797 KP000799 KP000800
KP000801 KP000802 KP000803 KP000804 KP000805 KP000806 KP000807 KP000808
KP000810 KP000813 KP000814 KP000815 KP000816 KP000817 KP000820 KP000825
KP000826 KP000827 KP000830 KP000833 KP000834 KP000835 KP000836 KP000837
KP000838 KP000839 JX545477 JX545478 JX545479 JX545480 JX545481 JX545482 JX545483
JX545484 JX545485 JX545486 JX545487 JX545488 JX545489 JX545490 JX545491 JX545492
JX545493 JX545494 JX545495 JX545496 JX545497 JX545498 JX545499 JX545500 JX545501
JX545502 JX545503 JX545504 JX545505 JX545506 JX545507 JX545508 JX545509 JX545510
JX545511 JX545512 JX545513 JX545514 JX545515 JX545516 JX545517 JX545518 JX545519
JX545520 JX545521 JX545522 JX545523 JX545524 JX545525 JX545526 JX545527 JX545528
JX545529 JX545530 JX545531 JX545532 JX545533 JX545534 JX545535 JX545536 JX545537
JX545538 JX545539 JX545540 JX545541 JX545542 JX545543 JX545544 JX545545 JX545546
JX545547 JX545548 JX545549 JX545550 JX545551 JX545552 JX545553 JX545554 JX545555
JX545556 JX545557 JX545558 JX545559 JX545560 JX545561 JX545562 JX545563 JX545564
JX545565 JX545566 JX545567 JX545568 JX545569 JX545570 JX545571 JX545572 JX545573
JX545574 JX545575 JX545576 JX545577 JX545578 JX545579 JX545580 JX545581 JX545582
JX545583 JX545584 JX545585 JX545586 JX545587 JX545588 JX545589 JX545590 JX545591
JX545592 JX545593 JX545594 JX545595 JX545596 JX545597 JX545598 JX545599 JX545600
JX545601 JX545602 JX545603 JX545604 JX545605 JX545606 JX545607 JX545608 JX545609
JX545610 JX545611 JX545612 JX545613 JX545614 JX545615 JX545616 JX545617 JX545618
JX545619 JX545620 JX545621 JX545622 JX545623 JX545624 JX545625 JX545626 JX545627
JX545628 JX545629 JX545630 JX545631 JX545632 JX545633 JX545634 JX545635 JX545636
JX545637 JX545638 JX545639 JX545640 JX545641 JX545642 JX545643 JX545644 JX545645
JX545646 JX545647 JX545648 JX545649 JX545650 JX545651 JX545652 JX545653 JX545654
JX545655 JX545656 JX545657 JX545658 JX545659 JX545660 JX545661 JX545662 JX545663
JX545664 JX545665 JX545666 JX545667 JX545668 JX545669 JX545670 JX545671 JX545672
JX545673 JX545674 JX545675 JX545676 JX545677 JX545678 JX545679 JX545680 JX545681
JX545682 JX545683 JX545684 JX545685 JX545686 JX545687 JX545688 JX545689 JX545690
JX545691 JX545692 JX545693 JX545694 JX545695 JX545696 JX545697 JX545698 JX545699
JX545700 JX545701 JX545702 JX545703 JX545704 JX545705 JX545706 JX545707 JX545708
JX545709 JX545710 JX545711 JX545712 JX545713 JX545714 JX545715 JX545716 JX545717
JX545718 JX545719 JX545720 JX545721 JX545722 JX545723 JX545724 JX545725 JX545726

JX545727 JX545728 JX545729 JX545730 JX545731 JX545732 JX545733 JX545734 JX545735 JX545736 JX545737 JX545738 JX545739 JX545740 JX545741 JX545742 JX545743 JX545744 JX545745 JX545746 JX545747 JX545748 JX545749 JX545750 JX545751 JX545752 JX545753 JX545754 JX545755 JX545756 JX545757 JX545758 JX545759 JX545760 JX545761 JX545762 JX545763 JX545764 JX545765 JX545766 JX545767 JX545768 JX545769 JX545770 JX545771 JX545772 JX545773 JX545774 JX545775 JX545776 JX545777 JX545778 JX545779 JX545780 JX545781 JX545782 JX545783 JX545784 JX545785 JX545786 JX545787 JX545788 JX545789 JX545790 JX545791 JX545792 JX545793 JX545794 JX545795 JX545796 JX545797 JX545798 JX545799 JX545800 JX545801 JX545802 JX545803 JX545804 JX545805 JX545806

***CYTB* gene (domestic sheep)**

JX545807 JX545808 JX545809 JX545810 JX545811 JX545812 JX545813 JX545814 JX545815 JX545816 JX545817 JX545818 JX545819 JX545820 JX545821 JX545822 JX545823 JX545824 JX545825 JX545826 JX545827 JX545828 JX545829 JX545830 JX545831 JX545832 JX545833 JX545834 JX545835 JX545836 JX545837 JX545838 JX545839 JX545840 JX545841 JX545842 JX545843 JX545844 JX545845 JX545846 JX545847 JX545848 JX545849 JX545850 JX545851 JX545852 JX545853 JX545854 JX545855 JX545856 JX545857 JX545858 JX545859 JX545860 JX545861 JX545862 JX545863 JX545864 JX545865 JX545866 JX545867 JX545868 JX545869 JX545870 JX545871 JX545872 JX545873 JX545874 JX545875 JX545876 JX545877 JX545878 JX545879 JX545880 JX545881 JX545882 JX545883 JX545884 JX545885 JX545886 JX545887 JX545888 JX545889 JX545890 JX545891 JX545892 JX545893 JX545894 JX545895 JX545896 JX545897 JX545898 JX545899 JX545900 JX545901 JX545902 JX545903 JX545904 JX545905 JX545906 JX545907 JX545908 JX545909 JX545910 JX545911 JX545912 JX545913 JX545914 JX545915 JX545916 JX545917 JX545918 JX545919 JX545920 JX545921 JX545922 JX545923 JX545924 JX545925 JX545926 JX545927 JX545928 JX545929 JX545930 JX545931 JX545932 JX545933 JX545934 JX545935 JX545936 JX545937 JX545938 JX545939 JX545940 JX545941 JX545942 JX545943 JX545944 JX545945 JX545946 JX545947 JX545948 JX545949 JX545950 JX545951 JX545952 JX545953 JX545954 JX545955 JX545956 JX545957 JX545958 JX545959 JX545960 JX545961 JX545962 JX545963 JX545964 JX545965 JX545966 JX545967 JX545968 JX545969 JX545970 JX545971 JX545972 JX545973 JX545974 JX545975 JX545976 JX545977 JX545978 JX545979 JX545980 JX545981 JX545982 JX545983 JX545984 JX545985 JX545986 JX545987 JX545988 JX545989 JX545990 JX545991 JX545992 JX545993 JX545994 JX545995 JX545996 JX545997 JX545998 JX545999 JX546000 JX546001 JX546002 JX546003 JX546004 JX546005 JX546006 JX546007 JX546008 JX546009 JX546010 JX546011 JX546012 JX546013 JX546014 JX546015 JX546016 JX546017 JX546018 JX546019 JX546020 JX546021 JX546022 JX546023 JX546024 JX546025 JX546026 JX546027 JX546028 JX546029 JX546030 JX546031 JX546032 JX546033 JX546034 JX546035 JX546036 JX546037 JX546038 JX546039 JX546040 JX546041 JX546042 JX546043 JX546044 JX546045 JX546046 JX546047 JX546048 JX546049 JX546050 JX546051 JX546052 JX546053 JX546054 JX546055 JX546056 JX546057 JX546058 JX546059 JX546060 JX546061 JX546062 JX546063 JX546064 JX546065 JX546066 JX546067 JX546068 JX546069 JX546070 JX546071 JX546072 JX546073 JX546074 JX546075 JX546076 JX546077 JX546078 JX546079 JX546080 JX546081 JX546082 JX546083 JX546084 JX546085 JX546086 JX546087 JX546088 JX546089 JX546090 JX546091 JX546092 JX546093 JX546094

JX546095 JX546096 JX546097 JX546098 JX546099 JX546100 JX546101 JX546102 JX546103 JX546104 JX546105 JX546106 JX546107 JX546108 JX546109 JX546110 JX546111 JX546112 JX546113 JX546114 JX546115 JX546116 JX546117 JX546118 JX546119 JX546120 JX546121 JX546122 JX546123 JX546124 JX546125 JX546126 JX546127 JX546128 JX546129 JX546130 JX546131 JX546132 JX546133

D-loop (wild sheep)

AF039579 AY091487 HM236184 KF677264 KF677267 KF677268 KF677271 KF938360 HM236186 HM236187 AY091489 AF039580 AY091490 AY091491 KF938361 KP402090 KP402091 KP402092 KP402094 KP402097 KP402100 KP402101 KP402104 KP402106 KP402107 HM236188 AF242347 AY091492 AF242348 KX609626 JX101654 JX673912 AY091493 AY091494 JN181255

***CYTB* gene (wild sheep)**

EU366057 EU366058 EU365977 EU365990 EU365985 EU366063 EU366065 EU366066 EU366067 EU366059 EU366060 EU366061 EU366062 EU365973 EU365986 EU365987 EU365979 EU365988 EU365991 EU365997 EU365998 EU366002 EU366003 EU366009 EU366040 EU366053 EU366068 EU366070 EU366073 EU365975 EU365976 EU366010 EU366016 EU366055 EU365978 EU365981 EU365982 EU366072 EU365983 EU365993 EU365994 EU365995 EU366005 EU366007 EU366008 EU366011 EU366012 EU366013 EU366022 EU366023 EU366025 EU366041 EU366054 EU366069 EU366045 EU366046 EU366050 EU366042 EU366052 EU366039 EU366043 EU366044 EU366017 EU366018 EU366026 EU366028 EU366029 EU366031 EU366032 EU366033 EU366035 EU366036 EU366038 EU366056 EU366071 EU366047 EU366048 EU366074 EU366049 EU365992 KY366509 KX910788 JX101654 FJ936213 FJ936200 FJ936181 FJ936188 FJ936217 FJ936182 FR873150 FR873152 FR873149 FJ936209 FJ936175 FJ936208 FJ936211 FJ936207 FJ936222 FJ936223 FJ936226 FJ936233 FJ936234 FJ936218 FJ936219 AJ867265 AJ867257 AJ867258 AJ867260 AJ867266 AJ867267 AJ867268 AJ867269 AJ867270 AJ867271 AJ867272 AJ867273 AJ867274 AJ867275 AJ867261 AJ867263 AJ867264 AJ867262 AF034729 AF034728 AF034727 AF242350 AF242349 HM236188 HM236186 HM236189 HM236185 U17860 U17859

Supplementary Table S2. Prior distributions of parameters for each tested scenario for Indian domestic sheep. N: effective population size t: time in generations μ: mean mutation rate.

	Parameters	Prior distribution	Parameters constrain
Lineage A			
Middle East effective population size	N_{ME}	Uniform (10^2,10^6)	
Mongolian Plateau effective population size	N_{MP}	Uniform (10^2,10^6)	
Indian Subcontinent effective population size	N_{IS}	Uniform (10^2,10^6)	
Time of the first divergence in the model	t1	Uniform (10^2,10^5)	
Time of the second divergence in the model	t2	Uniform (10^2,10^5)	t1>t2
Lineage B			
Middle East effective population size	N_{ME}	Uniform (10^2,10^6)	
Mongolian Plateau effective population size	N_{MP}	Uniform (10^2,10^6)	
Indian Subcontinent effective population size	N_{IS}	Uniform (10^2,10^6)	
Time of the first divergence in the model (scenario 1)	t1	Uniform (10^2,10^5)	
Time of the second divergence in the model (scenario 1)	t2	Uniform (10^2,10^5)	t1>t2
Divergence time of Mongolian Plateau from ME	t3	Uniform (10^2,10^5)	
Divergence time of Indian subcontinent from ME	t4	Uniform (10^2,10^5)	†
Mean mutation rate per generation for mitochondrial marker	μ*	Uniform (10^{-8}, 10^{-7})	

†We did not use constraint in this parameter because it is unknown, in lineage B, which population diverged first from the Middle East.

*Cornuet, J. M., Ravigné, A., & Estoup, A. (2010). Inference on population history and model checking using DNA sequence and microsatellite data with the software DIYABC (v1.0). *BMC Bioinformatics*, 11, 401.

Supplementary Table S3. Posterior parameter values for scenario with high probability in lineage A and B of Indian domestic sheep estimated using DIYABC. The unit time, counted in generation, was converted into years, based on an average generation time of one years (Lv et al. 2015).

		Posterior parameter estimation		
	Parameters	Median	95% CI	RMAE
Lineage A				
Middle East effective population size	N_{ME}	155000	21100 - 755000	0.588
Mongolian Plateau effective population size	N_{MP}	900000	583000 - 991000	0.215
Indian Subcontinent effective population size	N_{IS}	987000	951000 - 999000	0.100
Divergence time MP from IS	t1	11.5 ka	1.18 – 49.4 ka	0.673
Divergence time ME from IS	t2	45.9 ka	11.4 – 49.4 ka	0.388
Lineage B				
Middle East effective population size	N_{ME}	963000	865000 - 997000	0.112
Mongolian Plateau effective population size	N_{MP}	725000	31100 – 971000	0.222
Indian Subcontinent effective population size	N_{IS}	85900	517000 – 986000	0.191
Divergence time of Mongolian Plateau from ME	t3	22.2 ka	4.57 – 89.1	0.406
Divergence time of Indian subcontinent from ME	t4	35.3 ka	2.41 – 82.1 ka	0.498

N: effective population size t: time in years ka: thousand years RMAE: relative median of absolute error.

Lv, F. H. et al. (2015). Mitogenomic meta-analysis identifies two phases of migration in the history of eastern Eurasian sheep. *Mol. Biol. Evol.* 32, 2515-2533.
